# Endometriosis and uterine fibroids and risk of premature mortality: prospective cohort study

**DOI:** 10.1136/bmj-2023-078797

**Published:** 2024-11-20

**Authors:** Yi-Xin Wang, Leslie V Farland, Audrey J Gaskins, Siwen Wang, Kathryn L Terry, Kathryn M Rexrode, Janet W Rich-Edwards, Rulla Tamimi, Jorge E Chavarro, Stacey A Missmer

**Affiliations:** 1 Department of Environmental Health, School of Public Health, Shanghai Jiao Tong University, Shanghai, China; 2 Department of Nutrition, Harvard T.H. Chan School of Public Health, Boston, MA, USA; 3 Department of Epidemiology and Biostatistics, Mel and Enid Zuckerman College of Public Health and Department of Obstetrics and Gynecology, College of Medicine-Tucson, University of Arizona, Tucson, AZ, USA; 4 Department of Epidemiology, Rollins School of Public Health, Emory University, Atlanta, GA, USA; 5 Department of Epidemiology, Harvard T.H. Chan School of Public Health, Boston, MA, USA; 6 Obstetrics and Gynecology Epidemiology Center, Brigham and Women's Hospital and Harvard Medical School, Boston, MA, USA; 7 Division of Women’s Health, Department of Medicine, Brigham and Women’s Hospital, Harvard Medical School, Boston, MA, USA; 8 Channing Division of Network Medicine, Department of Medicine, Brigham and Women’s Hospital, Harvard Medical School, Boston, MA, USA; 9 Division of Epidemiology, Population Health Sciences, Weill Cornell Medicine, New York, NY, USA; 10 Department of Obstetrics and Gynecology and Reproductive Biology, College of Human Medicine, Michigan State University, Grand Rapids, MI, USA

## Abstract

**Objective:**

To prospectively assess the effect of endometriosis and uterine fibroids on the long term risk of premature mortality (younger than 70 years).

**Design:**

Prospective cohort study

**Setting:**

The Nurses’ Health Study II, United States (1989-2019).

**Participants:**

110 091 women aged 25-42 years in 1989 without a history of hysterectomy before endometriosis or fibroids diagnosis, cardiovascular diseases, or cancer.

**Main outcome measures:**

Hazard ratios (estimated by Cox proportional hazards models) for total and cause specific premature mortality according to laparoscopically confirmed endometriosis or ultrasound or hysterectomy confirmed uterine fibroids reported in biennial questionnaires.

**Results:**

4356 premature deaths were recorded during 2 994 354 person years of follow-up (27.2 years per person), including 1459 from cancer, 304 from cardiovascular diseases, and 90 from respiratory diseases. The crude incidence of all cause premature mortality for women with and without laparoscopically confirmed endometriosis was 2.01 and 1.40 per 1000 person years, respectively. In age adjusted models, laparoscopically confirmed endometriosis was associated with a hazard ratio of 1.19 (95% confidence interval 1.09 to 1.30) for premature death; these models were strengthened after also adjusting for potential confounders including behavioral factors (1.31, 1.20 to 1.44). Cause specific mortality analyses showed that the association was largely driven by mortality from senility and ill-defined diseases (1.80, 1.19 to 2.73), non-malignant respiratory diseases (1.95, 1.11 to 3.41), diseases of the nervous system and sense organs (2.50, 1.40 to 4.44), and malignant neoplasm of gynecological organs (2.76, 1.79 to 4.26). Ultrasound or hysterectomy confirmed uterine fibroids were not associated with all cause premature mortality (1.03, 0.95 to 1.11), but were associated with a greater risk of mortality from malignant neoplasm of gynecological organs (2.32, 1.59 to 3.40) in cause specific mortality analyses. The risk of mortality caused by cardiovascular and respiratory diseases varied according to joint categories of endometriosis and uterine fibroids, with an increased risk of all cause premature mortality among women reporting both endometriosis and uterine fibroids.

**Conclusion:**

Women with a history of endometriosis and uterine fibroids might have an increased long term risk of premature mortality extending beyond their reproductive lifespan. These conditions were also associated with an increased risk of death due to gynecological cancers. Endometriosis was associated with a greater risk of non-cancer mortality. These findings highlight the importance for primary care providers to consider these gynecological disorders in their assessment of women's health.

## Introduction

Two decades have passed since the United Nations launched the sustainable development goal of reducing premature mortality from non-communicable diseases by one third by 2030.^1^ However, all countries are still facing a huge burden of premature deaths from these diseases.^2^ The absolute number of deaths from non-communicable diseases between the ages of 30 and 69 years was estimated to be 15.6 million in 2019, accounting for 76% of overall premature deaths (20.4 million).^3^ ^4^ With the 2030 sustainable development goal target less than a decade away,^1^ identifying the risk factors of premature deaths from non-communicable diseases, particularly cancer, cardiovascular diseases, and respiratory diseases,^1^ is urgently needed to curb the growing burden of these diseases. Besides traditional risk factors affecting men and women, such as smoking, an unhealthy diet, and overweight or obesity,^5^ growing evidence shows that reproductive traits unique to women, such as menstrual cycle characteristics, gestational diabetes, gestational hypertension, and pregnancy loss, are associated with premature non-communicable disease mortality.^6^ ^7^ ^8^ ^9^ ^10^ ^11^

Endometriosis and uterine fibroids are common disorders among reproductive aged women, with a clinically relevant prevalence of 10% and 15-30%, respectively.^12^ ^13^ Endometriosis is characterized by growth outside the uterus of tissue resembling the endometrium. In contrast, uterine fibroids are non-malignant neoplasms made up of smooth muscle cells, typically growing within or around the myometrium. However, endometriosis and uterine fibroids share common genetic origins,^14^ and their development involves interacting endocrine, immunological, and proinflammatory processes.^12^ ^13^ Previous evidence shows that endometriosis and uterine fibroids are associated with a greater risk of cardiovascular disease,^15^ hypertension,^16^ ^17^ and cancer,^18^ ^19^ ^20^ suggesting a potential contribution to premature mortality.^21^ Although a few case-control and disease registry studies have explored the associations between endometriosis and uterine fibroids and the subsequent risk of total or cause specific mortality,^22^ ^23^ ^24^ these associations have not been examined in prospective cohort studies with careful control of potential confounding factors. Additionally, the influence of the co-occurrence of endometriosis and uterine fibroids needs to be assessed. More importantly, the potential modification effect of behavioral factors, hormone replacement therapy, hysterectomy or oophorectomy history, oral contraceptive use, and history of infertility, which is critical for improving preventive interventions, is also unclear. Therefore, we investigated the effect of endometriosis and uterine fibroids on the long term risk of premature mortality among women from the Nurses’ Health Study II (NHSII) in the United States, who have been followed biennially for three decades.

## Methods

### Study design

Our study was conducted within the NHSII, an ongoing prospective cohort started in 1989 by recruiting 116 429 US female nurses aged 25-42 years.^25^ Participants were followed biennially using mailed or electronic questionnaires that collected detailed data on reproductive traits, behavioral factors, and health status, with response rates in each follow-up cycle exceeding 90%. After excluding women who reported a history of physician diagnosed cardiovascular disease or cancer before enrollment (n=3067), had a hysterectomy before the diagnosis of endometriosis or uterine fibroids (n=3620), or reported endometriosis never confirmed by laparoscopy or uterine fibroids never confirmed by ultrasound or hysterectomy (n=349), 110 091 women were included in the present study (figure S1). The study protocol was approved by the institutional review boards of the Brigham and Women’s Hospital and Harvard T.H. Chan School of Public Health. Return of questionnaires indicated informed consent.

### Determining endometriosis and uterine fibroids

Starting in 1993 and biennially thereafter (figure S1), participants reported whether they ever had physician diagnosed endometriosis and uterine fibroids (uterine fibroids were updated until 2009 when most women had reached menopause). Participants who responded yes also reported the date of diagnosis and whether endometriosis and uterine fibroids were confirmed by laparoscopy, ultrasound, or hysterectomy. In 1993, the diagnosis date was reported as before September 1989, from September 1989 to May 1991, from June 1991 to May 1993, and after May 1993; these dates were used to identify the status of endometriosis and uterine fibroids in 1989 and 1991. Self-reported endometriosis was validated in 1994 (n=200) and 2011 (n=711), and the diagnosis of endometriosis was confirmed in the medical records of 95-100% of NHSII women reporting laparoscopically confirmed endometriosis, but in only 56% (15 of 27) of women without laparoscopic confirmation.^26^ In another subset of randomly selected NHSII participants who permitted a review of their medical records, the diagnosis of uterine fibroids was confirmed in 93% (108 of 116) of women reporting a diagnosis by ultrasound or hysterectomy.^27^ Therefore, in primary analyses we defined exposure based on laparoscopically confirmed endometriosis and ultrasound or hysterectomy confirmed uterine fibroids. Participants reporting endometriosis and fibroids that had not been clinically confirmed were not included in the analysis until the conditions were verified by laparoscopy, ultrasound, or hysterectomy in later follow-up cycles.

#### Assessment of covariates

Adult height, race or ethnicity, age at menarche, weight at age 18 years, and menstrual characteristics at ages 18-22 years were collected at baseline. Information on current weight, cigarette consumption, reproductive characteristics (eg, infertility history, pregnancy loss, and menopause status), night shiftwork duration, hormone replacement therapy, regular intake of aspirin, oral contraceptives, and non-aspirin non-steroidal anti-inflammatory drugs (NSAIDs, such as Aleve, Naprosyn, Relafen, ketoprofen, ibuprofen, and Anaprox; two or more times a week), and other health related factors (eg, hysterectomy and oophorectomy) were reported biennially since 1989. We calculated body mass index by dividing body weight (kilograms) by height (meters squared). Dietary intake, including alcohol consumption, was assessed every four years since 1991 using a validated semiquantitative food frequency questionnaire.^28^ ^29^ We computed the Alternate Healthy Eating Index 2010 score to reflect participants’ overall diet quality (0-110; a higher score indicates a healthier diet).^30^ Participants reported the average time of physical activities at baseline and every four years thereafter. We estimated the weekly hours spent on moderate to vigorous activities (eg, running, bicycling, and swimming).^11^ Self-reported body weight, behavioral factors, and reproductive characteristics have been mostly validated among participants from this cohort or the original Nurses’ Health Study.^7^ ^29^ ^31^ ^32^ ^33^ ^34^ ^35^ ^36^ ^37^ ^38^

### Determining premature mortality

We performed systematic searches of deaths for all participants from state vital statistics records and the National Death Index, supplemented by reports from next of kin or the postal authorities; these records were able to correctly identify more than 98% of deaths.^39^ Death causes were determined based on medical record review, autopsy reports, or death certificates, and then reviewed by a physician according to the eighth and ninth revisions of the international classification of diseases (table S1). We classified causes of death into 26 major categories according to the US Public Health Service, National Center for Health Statistics, and the Statistics Netherlands’ Database.^40^ ^41^ Deaths younger than 70 years were defined as premature mortality.^42^

### Statistical analysis

Person years of follow-up were calculated from the return date of the 1989 questionnaires until the end of follow-up (30 June 2019) or death, whichever occurred first (figure S1). Exposure status has been updated biennially since 1989 (uterine fibroids were updated until 2009). Participants were considered to have endometriosis or fibroids from the midpoint between the receipt of the previous questionnaire and the date on which the questionnaire with the first report was received. Therefore, a woman who did not have endometriosis or fibroids at recruitment and later developed one of these conditions contributed to exposure and non-exposure person years of follow-up. We estimated the hazard ratios and 95% confidence intervals for total and cause specific premature mortality according to visually confirmed endometriosis and uterine fibroids using Cox proportional hazard models. We also examined premature mortality risk according to the joint categories of endometriosis and uterine fibroids. To address potential confounding by age, calendar time, and their possible interactions, all Cox proportional hazard models were jointly stratified by age in months at the start of follow-up and the calendar year for the present survey cycle.^9^ The proportional assumption was tested by comparing models with and without multiplicative interaction terms between endometriosis or uterine fibroids and calendar time using the likelihood ratio test.^43^ Potential confounders were selected a priori based on previous findings of factors associated with premature mortality, endometriosis, and uterine fibroids and then determined by following the guidance from “Evidence synthesis for constructing directed acyclic graphs” (text S1 and figure S2). In the primary models, we adjusted for age, body mass index at age 18 years, menstrual cycle length at age 18-22 years, age at menarche, and time varying hormone replacement therapy and regular intake of non-aspirin NSAIDs, aspirin, and oral contraceptives. In the final models, we further adjusted for time varying body mass index, smoking status, physical activity, and Alternate Healthy Eating Index 2010 diet quality scores. To efficiently handle time varying covariates, the Anderson-Gill data structure was used to create new data records for each follow-up cycle at which participants were at risk, with covariates set to values at the time when follow-up questionnaires were received.^44^ Covariates with missing values at a given questionnaire cycle (mostly <5%; table 1) were carried forward using the most recent data; otherwise, missing indicators were created.^45^

**Table 1:** Age standardized baseline (1989) characteristics according to history of confirmed endometriosis and uterine fibroids diagnosis reported at baseline among 110 091 women (NHSII, 1989-2019)

Characteristics	Laparoscopically confirmed endometriosis at baseline			Ultrasound or hysterectomy confirmed uterine fibroids at baseline	
	No	Yes		No	Yes
Number of women	104 906	5185		105 010	5081
Age at recruitment (years), mean (SD)	34.61 (4.67)	36.06 (4.20)		34.53 (4.64)	37.88 (3.78)
Age at menarche (years)					
<12	25 338 (24)	1506 (30)		25 253 (24)	1591 (34)
12-13	60 631 (58)	2914 (56)		60 726 (58)	2819 (53)
>13	18 937 (18)	765 (15)		19 031 (18)	671 (13)
Body mass index at age 18					
<18.5	15 076 (14)	956 (18)		15 290 (15)	742 (15)
18.5-24.9	78 845 (75)	3815 (73)		78 870 (75)	3790 (72)
25-29.9	8303 (8)	316 (7)		8190 (8)	429 (9)
≥30	2682 (3)	98 (2)		2660 (3)	120 (3)
Body mass index at recruitment					
<18.5	3485 (3)	181 (4)		3557 (4)	109 (2)
18.5-24.9	66 521 (67)	3343 (67)		66 869 (67)	2995 (60)
25-29.9	18 103 (18)	949 (18)		17 954 (18)	1098 (21)
≥30	11 346 (11)	493 (10)		11 125 (11)	714 (16)
Moderate to vigorous physical activity at recruitment (hours/week), mean (SD)*	3.38 (5.05)	3.11 (4.71)		3.38 (5.05)	3.18 (4.70)
AHEI-2010 dietary score, mean (SD)†	47.99 (10.81)	47.08 (10.59)		47.96 (10.81)	47.69 (10.52)
Alcohol consumption at recruitment (g), mean (SD)	3.12 (6.05)	3.08 (6.49)		3.13 (6.07)	2.73 (5.29)
Parity at recruitment					
0	30 469 (29)	1907 (40)		31 265 (30)	1111 (27)
1	19 886 (19)	1213 (24)		20 126 (19)	973 (22)
2	34 764 (33)	1466 (26)		34 362 (33)	1868 (33)
≥3	19 787 (19)	599 (10)		19 257 (19)	1129 (18)
Non-Hispanic white	95 940 (93)	4835 (95)		96 296 (93)	4479 (88)
Current or former aspirin users at recruitment	11 272 (11)	705 (14)		11 289 (11)	688 (13)
Current or former non-aspirin NSAID users at recruitment	19 522 (19)	1410 (28)		19 744 (19)	1188 (24)
History of infertility at recruitment	17 059 (16)	2839 (52)		18 436 (18)	1462 (27)
Hysterectomy at recruitment	1413 (1)	1276 (21)		1317 (1)	1372 (20)
Oophorectomy at recruitment	668 (1)	1011 (17)		1008 (1)	671 (10)
Night shift work at recruitment	64 523 (62)	3320 (63)		64 754 (62)	3089 (63)
Long or irregular menstrual cycles in adulthood	14 946 (14)	652 (13)		15 011 (14)	587 (14)
Ever married at recruitment	90 434 (86)	4737 (88)		90 535 (86)	4636 (88)
Cigarette smoking status at recruitment					
Never	65 448 (65)	3128 (64)		65 472 (65)	3104 (62)
Former	21 030 (21)	1119 (21)		20 994 (21)	1155 (23)
Current	13 511 (14)	749 (15)		13 571 (14)	689 (15)
Oral contraceptive use at recruitment					
Never	18 266 (17)	568 (10)		18 114 (17)	720 (15)
Former	73 863 (71)	4230 (79)		73 949 (71)	4144 (77)
Current	12 777 (12)	387 (10)		12 947 (12)	217 (8)
Menstrual cycle length (days) at age 18-22 years					
<26	12 166 (12)	650 (13)		12 210 (12)	606 (12)
26-31	69 361 (66)	3478 (66)		69 454 (66)	3385 (65)
32-50	17 729 (17)	778 (15)		17 679 (17)	828 (19)
>50 or too irregular to estimate	5650 (5)	279 (6)		5667 (5)	262 (5)
Postmenopausal hormone therapy at recruitment					
Never	94 951 (91)	3378 (67)		94 479 (91)	3850 (80)
Former	7119 (7)	787 (16)		7326 (7)	580 (11)
Current	2144 (2)	957 (16)		2496 (2)	605 (10)
Menopause status at recruitment					
Premenopause	103 577 (99)	4325 (86)		103 317 (98)	4585 (92)
Postmenopause	713 (1)	814 (14)		1070 (1)	457 (7)
Uncertain	616 (1)	46 (1)		623 (1)	39 (1)

Data are numbers (%) unless stated otherwise. Means (standard deviation) for continuous variables and percentages for categorical variables are standardized to the age distribution of the study population, except for age.

AHEI=Alternative Healthy Eating Index; NSAID=non-steroidal anti-inflammatory drugs; SD=standard deviation.

Total of 5106 (4.6%), 5670 (5.2%), 2051 (1.9%), 755 (0.7%), and 24 076 (21.9%) women had missing data on baseline smoking status, body mass index, race, hormone replacement therapy, and diet (including alcohol intake), respectively.

* Estimated weekly time of doing moderate to vigorous activities that required at least three metabolic equivalent units per hour.

† AHEI-2010 score ranges from 0 (non-adherence) to 110 (perfect adherence); a higher score indicates a healthier diet.

To assess the competing risk across different death causes, competing risk Cox proportional hazards regression models were constructed to analyze the associations of endometriosis and uterine fibroids with cause specific mortality.^10^ ^46^ Given that unhealthy behavioral factors are strong risk factors of mortality,^46^ ^47^ we examined the effect modification for body mass index, diet quality, physical activity, and smoking status by classifying participants into low and high risk groups based on previous findings.^43^ ^47^ We also explored the effect modification for race or ethnicity, nulliparity, NSAID use, aspirin use, oral contraceptive use, spontaneous abortion, long or irregular menstrual cycles, postmenopausal hormone therapy, history of infertility, hysterectomy, and oophorectomy. Likelihood ratio tests were conducted to assess the multiplicative interaction between endometriosis and uterine fibroids and these stratification variables. Several sensitivity analyses were conducted.

We excluded participants without any follow-up questionnaires to evaluate potential bias resulting from loss of follow-up (n=1271).
In the analysis for endometriosis, we excluded women from the comparison group with a diagnosis of uterine fibroids (n=3995). Similarly, in the analysis for uterine fibroids, we excluded women with a history of endometriosis to assess if our findings were biased by the inclusion of the other disorder in the comparison group (n=4000).
To assess potential selection bias, we redefined mortality as deaths younger than 65 years (3646 deaths) or at any age (4480 deaths).^48^
We adjusted for the duration of rotating night shift work to assess the influence of night work.
We used baseline drug intake and behavioral factors to examine whether adjusting for time varying covariates affected the results.
We used the Markov chain Monte Carlo method of multiple imputations procedure to replace covariates with missing values to test the robustness of the carry forward method.
To minimize the risk of misdiagnosis between endometriosis or uterine fibroids and other neoplasms, we excluded women who died within five years of receiving a diagnosis of endometriosis or uterine fibroids (73 deaths).
We additionally adjusted for race or ethnicity, which could reflect the multigenerational and sociohistorical effects of racism and discrimination.^49^
Finally, we redefined endometriosis and uterine fibroids as all self-reported with and without confirmation by laparoscopy, ultrasound, or hysterectomy.

All data were analyzed using SAS 9.4 for UNIX (SAS Institute Inc., Cary, NC, USA). P values were false discovery rate adjusted when several tests were conducted simultaneously.^50^ To test potential unmeasured or uncontrolled confounding, we calculated E values using the publicly available online E value calculator.^51^ ^52^

### Patient and public involvement

No patients were involved in the initial design and implementation of the study because patient and public involvement was not common when the NHSII cohort was established. However, participants have offered valuable suggestions and comments throughout follow-up surveys, which have been integrated whenever possible. Additionally, we have taken into account suggestions and comments from an internal review panel comprising members of the public, as well as an advisory board consisting of nursing leaders. Dissemination to the public will include conference presentations, press releases, and plain language summaries shared on social media platforms.

## Results

Mean age of participants in 1989 and 2019 was 34.7±4.7 and 64.4±4.7 years, respectively. At baseline, women who reported laparoscopically confirmed endometriosis had a higher prevalence of infertility (age standardized percentages (unstandardized numerators and denominators): 52% (2839 of 5185) *v* 16% (17 059 of 104 906)), hysterectomy (21% (1276 of 5185) *v* 1% (1413 of 104 906)), and oophorectomy (17% (1011 of 5185) *v* 1% (668 of 104 906)), and were more likely to use non-aspirin NSAIDs (28% (1410 of 5185) *v* 19% (19 522 of 104 906)), and postmenopausal hormone therapy (32% (1744 of 5185) *v* 9% (9263 of 104 906)) compared with those without endometriosis. Similarly, slightly higher prevalences of infertility (27% (1462 of 5081) *v* 18% (18 436 of 105 010)), hysterectomy (20% (1372 of 5081) *v* 1% (1317 of 105 010)), and oophorectomy (10% (671 of 5081) *v* 1% (1008 of 105 010)), non-aspirin NSAID intake (24% (1188 of 5081)) *v* 19% (19 744 of 105 010)), and postmenopausal hormone therapy (21% (1185 of 5081) *v* 9% (9822 of 105 010)) at baseline were observed among women reporting ultrasound or hysterectomy confirmed uterine fibroids than those without fibroids (table 1).

**Fig 1:**
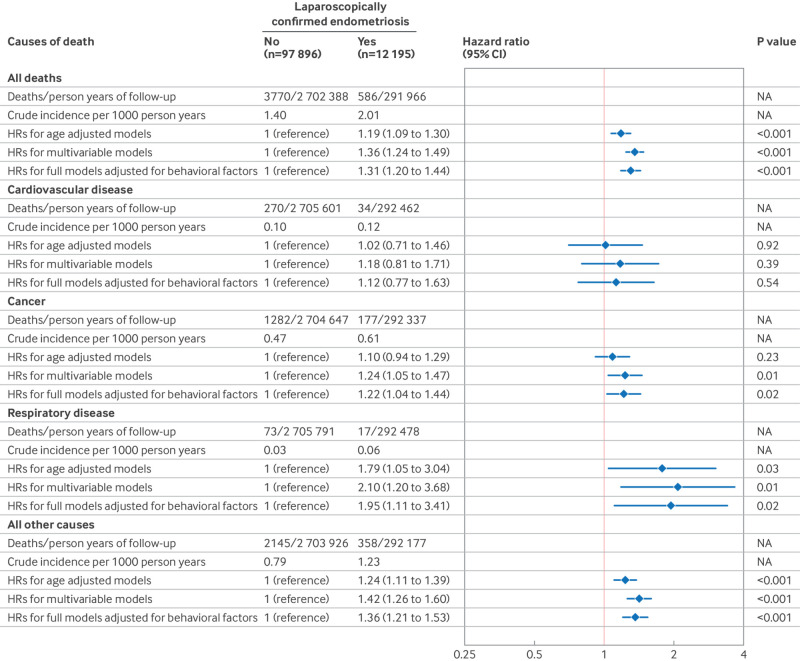
Risk of all cause and cause specific premature mortality (younger than 70 years) according to the occurrence of endometriosis among 110 091 women (Nurses’ Health Study II, 1989-2019). In age adjusted Cox proportional hazard regression models, analyses were stratified jointly by participants’ age in months at the start of follow-up and calendar years of the current questionnaire cycle. Multivariable models were further adjusted for history of infertility (yes, no (reference)), body mass index at age 18 years (<18.5, 18.5-24.9 (reference), 25-29.9, 20-34.9, ≥35), menstrual cycle length at age 18-22 years (<26, 26-31 (reference), 32-50, or ≥50 days or too irregular to estimate), age at menarche (<12 (reference), 12, 13, or ≥14 years), time varying postmenopausal hormone therapy (never (reference), former, current), non-aspirin non-steroidal anti-inflammatory drug use (yes, no (reference)), aspirin use (yes, no (reference)), and oral contraceptive use (current or former, no (reference)). Full models were further adjusted for time varying body mass index (<24.9 (reference), 25-29.9, 30-34.9, or ≥35), cigarette smoking status (never (reference), former, current 1-34 cigarettes/day, or current ≥35 cigarettes/day), physical activity (0 (reference), 0.1-1.0, 1.1-2.4, 2.5-5.9, or ≥6 h/week), and Alternative Healthy Eating Index 2010 diet quality scores (fifths, with lowest fifth (reference) representing least healthy diet). CI=confidence interval; HR=hazard ratio; NA=not applicable

**Fig 2:**
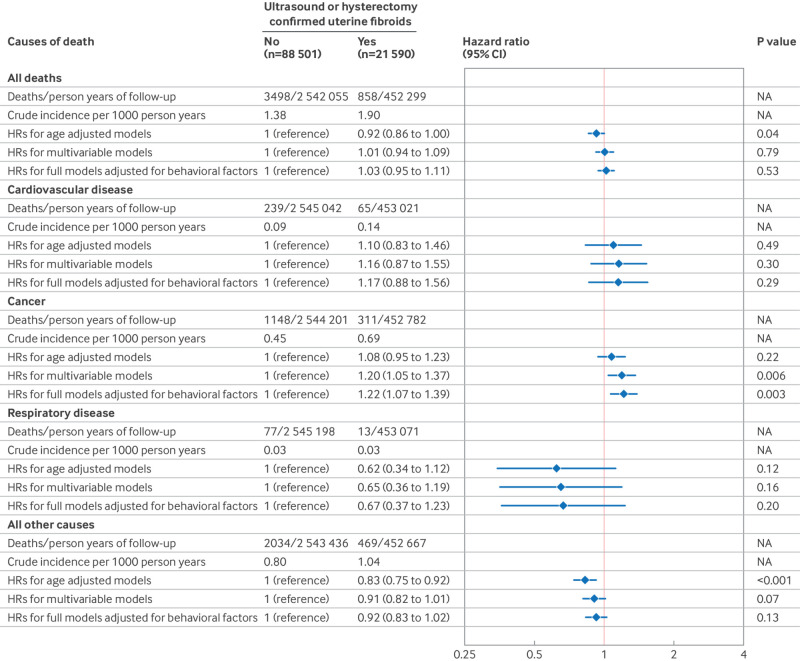
Risk of all cause and cause specific premature mortality (younger than 70 years) according to the occurrence of uterine fibroids among 110 091 women (Nurses’ Health Study II, 1989-2019). In age adjusted Cox proportional hazard regression models, analyses were stratified jointly by participants’ age in months at the start of follow-up and calendar years of the current questionnaire cycle. Multivariable models were further adjusted for history of infertility (yes, no (reference)), body mass index at age 18 years (<18.5, 18.5-24.9 (reference), 25-29.9, 20-34.9, ≥35), menstrual cycle length at age 18-22 years (<26, 26-31 (reference), 32-50, or ≥50 days or too irregular to estimate), age at menarche (<12 (reference), 12, 13, or ≥14 years), time varying postmenopausal hormone therapy (never (reference), past, current), non-aspirin non-steroidal anti-inflammatory drug use (yes, no (reference)), aspirin use (yes, no (reference)), and oral contraceptive use (current or former, no (reference)). Full models were further adjusted for time varying body mass index (<24.9 (reference), 25-29.9, 30-34.9, or ≥35), cigarette smoking status (never (reference), former, current 1-34 cigarettes/day, or current ≥35 cigarettes/day), physical activity (0 (reference), 0.1-1.0, 1.1-2.4, 2.5-5.9, or ≥6 h/week), and Alternative Healthy Eating Index 2010 diet quality scores (fifths, with lowest fifth (reference) representing least healthy diet). CI=confidence interval; HR=hazard ratio; NA=not applicable

**Fig 3:**
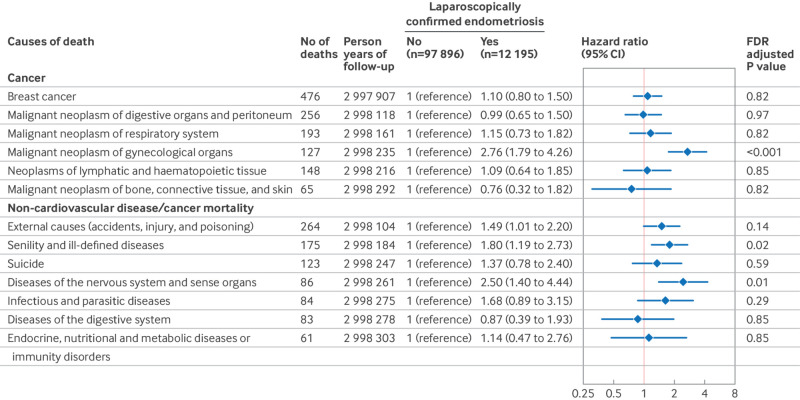
Risk of premature cause specific mortality (younger than 70 years) for less common causes of death according to the occurrence of endometriosis among 110 091 women (Nurses’ Health Study II, 1989-2019). Only conditions with 50 or more deaths are included. Models were adjusted for age (continuous), history of infertility (yes, no (reference)), body mass index at age 18 years (<18.5, 18.5-24.9 (reference), 25-29.9, 20-34.9, ≥35), menstrual cycle length at age 18-22 years (<26, 26-31 (reference), 32-50, or ≥50 days or too irregular to estimate), age at menarche (<12 (reference), 12, 13, or ≥14 years), time varying non-aspirin non-steroidal anti-inflammatory drug use (yes, no (reference)), aspirin use (yes, no (reference)), oral contraceptive use (current or former, no (reference)), postmenopausal hormone therapy (never (reference), past, current), body mass index (<24.9 (reference), 25-29.9, 30-34.9, or ≥35), smoking status (never (reference), former, current 1-34 cigarettes/day, or current ≥35 cigarettes/day), physical activity (0 (reference), 0.1-1.0, 1.1-2.4, 2.5-5.9, or ≥6 h/week), and Alternative Healthy Eating Index 2010 diet quality scores (fifths, with lowest fifth (reference) representing least healthy diet). CI=confidence interval; FDR=false discovery rate

**Fig 4:**
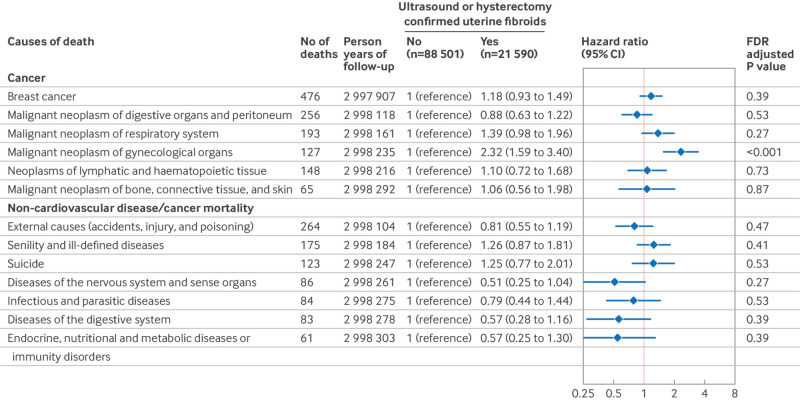
Risk of premature cause specific mortality (younger than 70 years) for less common causes of death according to the occurrence of uterine fibroids among 110 091 women (Nurses’ Health Study II, 1989-2019). Only conditions with 50 or more deaths are included. Models were adjusted for age (continuous), history of infertility (yes, no (reference)), body mass index at age 18 years (<18.5, 18.5-24.9 (reference), 25-29.9, 20-34.9, ≥35), menstrual cycle length at age 18-22 years (<26, 26-31 (reference), 32-50, or ≥50 days or too irregular to estimate), age at menarche (<12 (reference), 12, 13, or ≥14 years), and time varying non-aspirin non-steroidal anti-inflammatory drug use (yes, no (reference)), aspirin use (yes, no (reference)), oral contraceptive use (current or former, no (reference)), postmenopausal hormone therapy (never (reference), past, current), body mass index (<24.9 (reference), 25-29.9, 30-34.9, or ≥35), smoking status (never (reference), former, current 1-34 cigarettes/day, or current ≥35 cigarettes/day), physical activity (0 (reference), 0.1-1.0, 1.1-2.4, 2.5-5.9, or ≥6 h/week), and Alternative Healthy Eating Index 2010 diet quality scores (fifths, with lowest fifth (reference) representing least healthy diet). CI=confidence interval; FDR=false discovery rate

During 2 994 354 person years of follow-up (27.2 years per person), 11.0% (12 195 of 110 091) of women reported laparoscopically confirmed endometriosis (fig 1), and 19.6% (21 590 of 110 091) of women reported ultrasound or hysterectomy confirmed uterine fibroids at baseline or during follow-up (fig 2). In total, we documented 4356 premature deaths, including 1459 from cancer, 304 from cardiovascular diseases, and 90 from respiratory diseases (table S1). The crude incidence of all cause, premature mortality for women with and without laparoscopically confirmed endometriosis was 2.01 and 1.40 per 1000 person years, respectively. In age adjusted models, laparoscopically confirmed endometriosis was associated with a hazard ratio of 1.19 (95% confidence interval 1.09 to 1.30) for premature death (fig 1). These associations became stronger after also adjusting for potential confounders, including behavioral factors (1.31, 1.20 to 1.44; fig 1). Cause specific mortality analyses revealed that laparoscopically confirmed endometriosis was associated with a greater risk of mortality from cancer (1.22, 1.04 to 1.44) and respiratory diseases (1.95, 1.11 to 3.41; fig 1). With further in-depth analyses examining causes of death (only including categories with more than 50 deaths), laparoscopically confirmed endometriosis was associated with a greater risk of mortality caused by senility and ill-defined diseases (1.80, 1.19 to 2.73), diseases of the nervous system and sense organs (2.50, 1.40 to 4.44), and malignant neoplasm of gynecological organs (2.76, 1.79 to 4.26; fig 3). Uterine fibroids were unrelated to all cause premature mortality (1.03, 0.95 to 1.11; fig 2). However, we observed an increased cancer mortality risk among women reporting ultrasound or hysterectomy confirmed uterine fibroids (1.22, 1.07 to 1.39; fig 2), primarily driven by malignant neoplasm of gynecological organs (2.32, 1.59 to 3.40; fig 4).

When we jointly categorized participants by occurrence of endometriosis and uterine fibroids (table S2), we found an increased risk of total mortality among women reporting endometriosis only (1.32, 1.19 to 1.47) and those reporting both endometriosis and uterine fibroids (1.31, 1.12 to 1.53). In cause specific mortality analyses, similar cancer mortality risks were observed among women reporting endometriosis only (1.34, 1.10 to 1.62), uterine fibroids only (1.28, 1.11 to 1.47), and both conditions (1.20, 0.90 to 1.61). The adjusted hazard ratio of cardiovascular disease mortality among women reporting both endometriosis and uterine fibroids was 1.61 (0.93 to 2.76), 0.93 (0.57 to 1.51) among women reporting endometriosis only, and 1.07 (0.78 to 1.48) among those with uterine fibroids only. Additionally, an increased risk of respiratory disease mortality was exclusively observed among women reporting endometriosis only (2.21, 1.19 to 4.10).

Given the limited numbers of deaths from cardiovascular diseases and respiratory diseases, stratified analyses were only conducted for total and cancer mortality. We found no convincing effect modification by race or ethnicity, diet quality, cigarette smoking status, body mass index, physical activity, postmenopausal hormone therapy, history of infertility, oophorectomy, aspirin use, non-aspirin NSAID use, spontaneous abortion history, long or irregular menstrual cycles, and oral contraceptive use (all P for interaction (false discovery rate adjusted) >0.10; table 2 and table S3). However, we found that uterine fibroids were associated with a lower risk of total and cancer premature mortality among women with hysterectomy (P for interaction (false discovery rate adjusted)=0.09 and 0.08, respectively; table 2 and table S3). We also observed a stronger association between uterine fibroids and cancer mortality among women who were nulliparous (P for interaction (false discovery rate adjusted)=0.06; table S3). The associations between endometriosis and uterine fibroids and premature mortality persisted in competing risk Cox proportional hazards regression models (tables S4 and S5) and were materially unchanged in several sensitivity analyses assessing the influence of non-response to follow-up questionnaires, selection bias, race or ethnicity, diagnostic bias, and potential confounders (tables S6-S17). The E values were 1.95, 1.74, and 3.31 for all cause, cancer, and respiratory disease mortality, respectively, relating to endometriosis, and 1.74 for cancer mortality relating to uterine fibroids (table S18).

**Table 2:** Hazard ratio (95% confidence interval) for risk of all cause premature mortality (younger than 70 years) according to confirmed endometriosis and uterine fibroids diagnosis among 110 091 women, stratified by behavioral and reproductive factors (NHSII, 1989-2019)

Stratified factors	Laparoscopically confirmed endometriosis			Ultrasound or hysterectomy confirmed uterine fibroids	
	No	Yes		No	Yes
**Race or ethnicity**					
Non-Hispanic white (3912 deaths)	1.00 (reference)	1.33 (1.21 to 1.46)		1.00 (reference)	1.03 (0.95 to 1.12)
Other (444 deaths)	1.00 (reference)	1.23 (0.89 to 1.71)		1.00 (reference)	0.95 (0.74 to 1.22)
P for interaction	0.86	—		0.43	—
**Diet quality**					—
Top 40% (1116 deaths)	1.00 (reference)	1.35 (1.13 to 1.62)		1.00 (reference)	1.12 (0.97 to 1.29)
Bottom 60% (3240 deaths)	1.00 (reference)	1.29 (1.15 to 1.43)		1.00 (reference)	0.99 (0.90 to 1.08)
P for interaction	0.96	—		0.75	—
**Smoking status**					
Never smokers (2178 deaths)	1.00 (reference)	1.28 (1.12 to 1.47)		1.00 (reference)	0.98 (0.87 to 1.09)
Current or former smokers (2178 deaths)	1.00 (reference)	1.38 (1.22 to 1.57)		1.00 (reference)	1.07 (0.96 to 1.20)
P for interaction	0.83	—		0.75	—
**Body mass index**					
<25 (1793 deaths)	1.00 (reference)	1.22 (1.05 to 1.41)		1.00 (reference)	1.10 (0.96 to 1.25)
≥25 (2563 deaths)	1.00 (reference)	1.37 (1.22 to 1.53)		1.00 (reference)	0.99 (0.89 to 1.09)
P for interaction	0.83	—		0.68	—
**Physical activity (min/day)**					
≥30 (1310 deaths)	1.00 (reference)	1.52 (1.28 to 1.80)		1.00 (reference)	1.04 (0.90 to 1.21)
<30 (3046 deaths)	1.00 (reference)	1.24 (1.11 to 1.39)		1.00 (reference)	1.02 (0.93 to 1.12)
P for interaction	0.83	—		0.75	—
**Postmenopausal hormone therapy**					
No (2305 deaths)	1.00 (reference)	1.17 (0.97 to 1.40)		1.00 (reference)	1.09 (0.96 to 1.23)
Yes (2051 deaths)	1.00 (reference)	1.26 (1.13 to 1.41)		1.00 (reference)	0.93 (0.84 to 1.02)
P for interaction	0.83	—		0.15	—
**Parity**					
Nulliparous (1235 deaths)	1.00 (reference)	1.17 (0.99 to 1.38)		1.00 (reference)	1.08 (0.93 to 1.25)
Parous (3121 deaths)	1.00 (reference)	1.34 (1.20 to 1.49)		1.00 (reference)	1.00 (0.91 to 1.09)
P for interaction	0.53	—		0.83	—
**History of infertility**					
No (3247 deaths)	1.00 (reference)	1.34 (1.19 to 1.51)		1.00 (reference)	1.03 (0.94 to 1.13)
Yes (1109 deaths)	1.00 (reference)	1.28 (1.11 to 1.47)		1.00 (reference)	1.01 (0.87 to 1.17)
P for interaction	0.86	—		0.99	—
**History of hysterectomy**					
No (3627 deaths)	1.00 (reference)	1.05 (0.91 to 1.21)		1.00 (reference)	0.96 (0.86 to 1.06)
Yes (729 deaths)	1.00 (reference)	1.38 (1.19 to 1.60)		1.00 (reference)	0.74 (0.63 to 0.86)
P for interaction	0.23	—		0.09	—
**History of oophorectomy**					
No (792 deaths)	1.00 (reference)	1.18 (1.02 to 1.37)		1.00 (reference)	0.96 (0.83 to 1.12)
Yes (3564 deaths)	1.00 (reference)	1.14 (0.99 to 1.31)		1.00 (reference)	0.94 (0.85 to 1.04)
P for interaction	0.96	—		0.80	—
**Asprin use**					
Never (3425 deaths)	1.00 (reference)	1.30 (1.17 to 1.45)		1.00 (reference)	0.99 (0.90 to 1.08)
Current or former (931 deaths)	1.00 (reference)	1.28 (1.07 to 1.52)		1.00 (reference)	1.15 (0.99 to 1.33)
P for interaction	0.96	—		0.75	—
**Non-aspirin NSAID use**					
Never (3030 deaths)	1.00 (reference)	1.30 (1.16 to 1.45)		1.00 (reference)	0.99 (0.90 to 1.09)
Current or former (1326 deaths)	1.00 (reference)	1.31 (1.12 to 1.52)		1.00 (reference)	1.08 (0.95 to 1.23)
P for interaction	0.86	—		0.97	—
**Spontaneous abortion history**					
No (3526 deaths)	1.00 (reference)	1.33 (1.20 to 1.47)		1.00 (reference)	1.04 (0.95 to 1.13)
Yes (830 deaths)	1.00 (reference)	1.23 (1.01 to 1.49)		1.00 (reference)	0.98 (0.83 to 1.16)
P for interaction	0.83	—		0.92	—
**Long or irregular menstrual cycles in adulthood**					
No (3681 deaths)	1.00 (reference)	1.34 (1.21 to 1.47)		1.00 (reference)	1.03 (0.94 to 1.12)
Yes (675 deaths)	1.00 (reference)	1.14 (0.89 to 1.46)		1.00 (reference)	1.01 (0.83 to 1.23)
P for interaction	0.83	—		0.99	—
**Oral contraceptive use**					
Never (1195 deaths)	1.00 (reference)	1.25 (1.00 to 1.56)		1.00 (reference)	1.01 (0.85 to 1.21)
Current or former (3161 deaths)	1.00 (reference)	1.31 (1.19 to 1.44)		1.00 (reference)	1.02 (0.94 to 1.11)
P for interaction	0.83	—		0.75	—

Models were adjusted for age (continuous), race or ethnicity (non-Hispanic white, other (reference)), history of infertility (yes, no (reference)), body mass index at age 18 years (<18.5, 18.5-24.9 (reference), 25-29.9, 20-34.9, ≥35), menstrual cycle length at age 18-22 years (<26, 26-31 (reference), 32-50, or ≥50 days or too irregular to estimate), age at menarche (<12 (reference), 12, 13, or ≥14 years of age), time varying non-aspirin NSAID use (yes, no (reference)), aspirin use (yes, no (reference)), oral contraceptive use (current or former, no (reference)), postmenopausal hormone therapy (never (reference), former, current), body mass index (<24.9 (reference), 25-29.9, 30-34.9, or ≥35), smoking status (never (reference), former, current 1-34 cigarettes/day, or current ≥35 cigarettes/day), physical activity (0 (reference), 0.1-1.0, 1.1-2.4, 2.5-5.9, or ≥6 h/week), and Alternative Healthy Eating Index 2010 diet quality score (fifths), with lowest fifth (reference) representing the least healthy diet), excluding the stratifying variable. P values were false discovery rate adjusted.

## Discussion

### Principal findings

Results from this large prospective cohort showed that visually confirmed endometriosis and uterine fibroids were associated with a greater long term risk of premature mortality, driven primarily by malignant neoplasm of gynecological organs. Additionally, endometriosis was associated with a greater risk of non-malignant mortality caused by respiratory disease, senility and ill-defined diseases, and diseases of the nervous system and sense organs.

### Comparison with other studies

Few studies to date have explored the long term influence of endometriosis or uterine fibroids on mortality. In contrast to our findings, Saavalainen and colleagues reported a lower risk of mortality from all causes, cardiovascular diseases, cancer, accidents and violence, and respiratory diseases among 49 956 women with surgically verified endometriosis compared with a reference cohort of 98 824 age and municipality matched women^22^; and Shen and colleagues reported a lower risk of mortality from breast cancer in 22 001 women with a diagnosis of uterine fibroids compared with 85 356 women who were fibroid free matched by age and date of diagnosis.^23^ These previous studies did not determine gynecological diseases during follow-up or throughout the reproductive lifespan for participants in the reference group. Considering that women who did not report endometriosis or uterine fibroids at recruitment might develop these disorders during follow-up or in later life, misclassification should be taken into account if women with undiagnosed endometriosis or uterine fibroids are included among the population matched controls. Additionally, these retrospective case-control studies used register based data, which did not collect data on relevant confounders (eg, oral contraceptives, hormone replacement therapy, and infertility) and behavior factors (eg, body mass index, diet quality, physical activity, and smoking status), which might have resulted in biased associations. In support of this notion, strengthened hazard ratios were observed when we adjusted for these important covariates. Finally, the differences in population characteristics (eg, parity and professions) and their access to medical care and treatment resources might also lead to inconsistent findings between studies. In our present study, uterine fibroids were associated with a lower risk of total and cancer premature mortality among women with hysterectomy. We hypothesize that hysterectomy might have eliminated the potential for later life fibroid diagnosis and the development of certain malignancies, consequently reducing the risk of cancer mortality. Furthermore, we observed a stronger association between uterine fibroids and cancer mortality among women who were nulliparous, which is also plausible given the extensive evidence showing that nulliparity is associated with an increased risk of gynecological malignancies, such as ovarian and endometrial cancer.^53^ ^54^ However, the associations between endometriosis and uterine fibroids and total and cancer mortality did not vary by any behavior factors, indicating that these associations were independent of body mass index, diet quality, physical activity, and smoking status.

In support of our findings, several studies have shown that endometriosis is associated with a greater risk of cancer from gynecological organs such as ovarian, tubal, and endometrial cancer.^20^ ^55^ ^56^ ^57^ ^58^ ^59^ ^60^ Other studies also reported an association of uterine fibroids with a greater risk of ovarian and endometrial cancer.^58^ ^59^ Endometriosis and uterine fibroids are strong drivers of female infertility.^61^ ^62^ ^63^ The present findings are also consistent with our recent study from the same cohort (NHSII; n=101 777), in which infertility caused by ovulatory disorders and endometriosis was associated with a greater risk of premature mortality caused by all cancers and gynecological cancers.^7^ Breast cancer is the second leading cause of cancer deaths among US women.^64^ In our present study, we did not find any evidence of associations between endometriosis and breast cancer mortality, which is consistent with the findings of our previous study showing that endometriosis is unrelated to the overall risk of breast cancer among 116 430 NHSII women.^65^

Laparoscopically confirmed endometriosis has been associated with a higher risk of early onset coronary heart disease and stroke among women from the NHSII.^15^ ^66^ In our present study, endometriosis was unrelated to premature cardiovascular disease mortality, which could be partly explained by the low number of cardiovascular disease deaths in women who might not have reached 70 years of age. However, when we jointly categorized participants by exposure to endometriosis and uterine fibroids, an increased risk of cardiovascular disease mortality was observed among women reporting both endometriosis and uterine fibroids, albeit the confidence interval crossing the null value, but not among women with endometriosis only or uterine fibroids only. This finding suggests that endometriosis might interact synergistically with uterine fibroids, possibly accelerating the risk of cardiovascular disease mortality in later life. Conversely, we observed an increased risk of respiratory disease mortality exclusively among women reporting endometriosis only. However, similar cancer mortality risks were observed among women reporting endometriosis only, uterine fibroids only, and both conditions. These findings highlight the intricate interplay between endometriosis, uterine fibroids, and mortality, suggesting that different combinations of these conditions might present varying risks for different causes of mortality. Further research is warranted to unravel underlying mechanisms and investigate potential preventive and therapeutic interventions.

### Underlying mechanisms of observed associations

The associations of visually confirmed endometriosis and uterine fibroids with cancer mortality, particularly deaths caused by malignant neoplasm of gynecological organs, might reflect shared mechanistic pathways (eg, hyperestrogenism, oxidative stress, and inflammation) that synergistically contribute to these gynecological diseases and cancer mortality. For example, emerging evidence from animals and humans supports the distinct roles of estrogen and progesterone in the pathogenesis of endometrial cancer, endometriosis, and uterine fibroids.^67^ Meanwhile, many studies show that dysregulation of immune and inflammatory responses plays an important part in the pathology of various endometrial disorders, including endometriosis,^68^ ^69^ ^70^ uterine fibroids,^71^ and endometrial or ovarian cancer.^72^ ^73^ The associations of endometriosis and uterine fibroids with cancer mortality might also be partly explained by shared genetic factors,^74^ ^75^ indicating potential causal associations. For instance, animal and human studies have shown that endometriosis and endometrial cancer share numerous genes, including certain genes located within the “endometrial cancer pathway” such as PTEN, PTPRD, and ARID1A.^76^ ^77^ ^78^ In a recent large scale genome wide association study, Kho and colleagues reported a potential causal association between uterine fibroids and endometrial cancer in the Mendelian randomization analysis and identified several shared genetic risk regions between endometriosis and uterine fibroids and endometrial cancer.^79^

Diagnostic bias must be considered, particularly for the association with premature mortality caused by malignant neoplasm of gynecological organs. As observed when temporal rigour is applied to studies of endometriosis and endometrial cancer risk,^20^ endometriosis, which is marked by lengthy diagnostic delays partly because of missed diagnoses and misdiagnoses,^80^ might be detected only during investigating symptoms that are driven by the malignant condition. Although the present study applied a rigorous prospective design and only 73 premature deaths occurred within five years of receiving a diagnosis of endometriosis or uterine fibroids, it is possible that the diagnosis was, years before death, driven by evaluation for symptoms that subsequently were attributed to the gynecological malignancy, which would later be found and caused premature death, therefore inflating the magnitude of the risk estimations.

The mechanisms underlying the positive associations between endometriosis and mortality caused by respiratory disease, senility and ill-defined diseases (eg, debility and headache), and diseases of the nervous system and sense organs (eg, diseases of the central nervous system, nerves, and peripheral ganglia) are less straightforward to hypothesize. However, data are emerging that women with endometriosis might have a longer and potentially more severe SARS-CoV-2 infection,^81^ while associations with asthma and other heightened allergic responses are documented^82^; these might support pathways by which greater premature mortality from respiratory diseases is plausible. In a recent meta-analysis consisting of nine studies with 287 174 participants, endometriosis was associated with an increased risk of migraine headaches.^83^ Endometriosis and migraine headaches have shared symptoms and pathophysiological pathways.^83^ For instance, the activation of sensory fibers within ectopic endometrial tissue, along with an excessive number of activated and degranulating mast cells in endometriosis lesions or internal nerve structures, might trigger the release of algesic and proinflammatory mediators.^84^ ^85^ This process can sensitize primary afferent meningeal nociceptive neurons, leading to hyperalgesia and hypersensitivity, and ultimately might result in headaches. Death from diseases of the nervous system, such as inflammatory diseases of the central nervous system, could partly be explained by neuroinflammation and a greater risk of nociplastic pain and multisystemic pain related conditions observed in women with endometriosis.^86^ ^87^ However, additional studies with more premature deaths are needed to verify our findings and explore underlying mechanisms.

### Strengths and limitations

The strengths of our study include the large population size, longitudinal design with excellent response rates, sufficient numbers of premature deaths, and detailed collection of various potential confounders and behavior factors updated as frequently as every two years. In addition, participants were followed up biennially across most of their reproductive lifespan, which reduced the potential errors in the recall of endometriosis and uterine fibroids.

However, some important limitations should also be considered. Endometriosis and uterine fibroids were self-reported, which could result in exposure misclassification. This misclassification, although likely to be non-differential with respect to mortality owing to our longitudinal study design, could have biased our estimations. However, we defined women with gynecological conditions as those who reported visually confirmed endometriosis and uterine fibroids that were previously validated against medical records in the present cohort with extremely high reporting accuracy.^27^ ^88^ Additionally, more than 90% of our study participants were non-Hispanic white women and all had relatively homogenous professions and educational attainments, which might hamper the generalizability of our results to other racial or ethnic groups, particularly those who might face greater barriers accessing medical care and treatment resources.

As with any observational study, we can infer but not determine causal associations; neither endometriosis nor uterine fibroids could be experimentally assigned. Despite statistical control for various confounders and behavior factors, residual confounding cannot be entirely excluded. However, our estimated E values showed that an unmeasured confounder would need to be associated with endometriosis, uterine fibroids, and mortality by a magnitude of at least 1.74-fold, beyond the measured confounders in adjusted Cox models, to explain away any positive associations observed. Therefore, it is unlikely that unmeasured or unknown confounders would fully explain away our findings. Furthermore, we could not entirely rule out Collider stratification bias caused by adjusting for time varying confounders (eg, drug intake and body mass index) that might share common causes with outcomes.^89^ Additionally, the limited number of deaths other than cancers might be insufficient for precise estimations. Finally, in this large, geographically diverse cohort observed over three decades, we did not collect data on biopsy confirmed endometriosis pathology, lesion location, adenomyosis status, types of uterine fibroids (by ultrasound and hysterectomy), and certain drug intakes (eg, pain relievers), which might have hindered the precision of risk estimations.

### Conclusions

In this extensive longitudinal study of nurses tracked biennially over three decades, we found that visually confirmed endometriosis and uterine fibroids were associated with a greater long term risk of premature mortality, driven primarily by malignant neoplasm of gynecological organs. Endometriosis was also associated with a greater risk of non-cancer mortality. Our results suggest that women with a history of endometriosis and uterine fibroids might have an increased long term risk of premature mortality extending beyond their reproductive lifespan. These findings highlight the importance for primary care providers to consider both conditions in their assessment of women's health.

**What is already known on this topic**

Growing evidence shows that endometriosis and uterine fibroids are associated with a greater long term risk of chronic diseases, but the effect of endometriosis and uterine fibroids on premature mortality risk (death younger than 70 years) remains unclear

**What this study adds**

Results from this large prospective cohort suggest that women with visually confirmed endometriosis and uterine fibroids might have a greater long term risk of premature mortality extending beyond their reproductive lifespan
These conditions were also associated with an increased risk of death due to gynecological cancers
Endometriosis was associated with a greater risk of non-cancer mortality, highlighting the importance for primary care providers to consider these gynecological disorders in their assessment of women's health

## Data Availability

The data used in the present study will not be made publicly available, but they are accessible by contacting the research staff from NHSII at https://www.nurseshealthstudy.org/researchers. The analytic SAS codes are available from the corresponding author and can also be found in supporting materials.

## Web extra

Extra material supplied by authors

Web appendix 1: Supplemental material

## Notes

Contributors: Y-XW analyzed and drafted the original manuscript. SAM, JEC, and LVF contributed to funding acquisition and project administration. LVF checked the accuracy of data and analysis codes. All authors participated in the interpretation of the results and critical revision of the manuscript. The corresponding author attests that all listed authors meet authorship criteria and that no others meeting the criteria have been omitted. Y-XW and SAM are guarantors.

Transparency: The lead author affirms that the manuscript is an honest, accurate, and transparent account of the study being reported; that no important aspects of the study have been omitted; and that any discrepancies from the study as planned (and, if relevant, registered) have been explained.

Dissemination to participants and related patient and public communities: Dissemination to the public will include conference presentations, press releases, and plain language summaries shared on social media platforms. Additionally, NHSII participants will be periodically updated through various channels, including our study website and yearly newsletters.

Provenance and peer review: Not commissioned; externally peer reviewed.
