# Supplementary material for: Endometriosis and uterine fibroids and risk of premature mortality: prospective cohort study
Source: BMJ. 2024 Nov 20;387:e078797. doi: 10.1136/bmj-2023-078797 (PMC11577545; doi:10.1136/bmj-2023-078797)
Supplement: Supplementary file 1 — Web appendix 1: Supplemental material [file wany078797.ww.pdf]

SUPPLEMENTAL MATERIAL

Endometriosis and Uterine Fibroids and Risk of Premature Mortality: The Nurses' Health Study II

Yi-Xin Wang, Leslie V. Farland, Audrey J. Gaskins, Siwen Wang, Kathryn L Terry, Kathryn M Rexrode, Janet W. Rich-Edwards, Rulla Tamimi, Jorge E. Chavarro, and Stacey A. Missmer

Figure S1. Study design and exclusion criteria.

Table S2. Categories for causes of death.

Text S1. The process of constructing a directed acyclic graph (DAG) and determining potential confounders.

Table S2. Hazard ratio (HR) (95% confidence interval (CI)) for the risk of all-cause and cause-specific premature mortality (before age 70 y) according to the joint occurrence of endometriosis and uterine fibroids among 110,091 women (n=110,091; NHSII, 1989-2019).

Table S3. Hazard ratio (HR) (95% confidence interval (CI)) for the risk of premature cancer mortality according to confirmed endometriosis and uterine fibroids diagnosis among 110,091 women, stratified by behavioural and reproductive factors (n=110,091; NHSII, 1989-2019).

Table S4. Hazard ratio (HR) (95% confidence interval (CI)) for the risk of cause-specific mortality in relation to laparoscopically confirmed endometriosis based on competing-risk regression models (n=110,091; NHSII, 1989-2019).

Table S5. Hazard ratio (HR) (95% confidence interval (CI)) for the risk of cause-specific mortality in relation to uterine fibroids based on competing-risk regression models (n=110,091; NHSII, 1989-2019).

Table S6. Sensitivity analyses for the association of endometriosis and uterine fibroids with the risk of premature mortality by excluding women who never returned follow-up questionnaires (N=108,820; NHSII, 1989-2019).

Table S7. Sensitivity analyses for the association of endometriosis and uterine fibroids with the risk of premature mortality using the Markov chain Monte Carlo method of multiple imputations procedure to replace covariates with missing values (n=110,091; NHSII, 1989-2019).

Table S8. Sensitivity analyses for the association of endometriosis and uterine fibroids with the risk of premature mortality, with additional adjustment for night shift work (n=110,091; NHSII, 1989-2019).

Table S9. Sensitivity analyses for the association of endometriosis with the risk of premature mortality by excluding women from the comparison group who had uterine fibroids (n=106,096; NHSII, 1989-2019).

Table S10. Sensitivity analyses for the association of uterine fibroids with the risk of premature mortality by excluding women from the comparison group who had endometriosis (n=106,091; NHSII, 1989-2019).

Table S11. Sensitivity analyses for the association of endometriosis and uterine fibroids with risk of premature mortality by redefined premature mortality as deaths before 65 years of age (NHSII, 1989-2019).

Table S12. Sensitivity analyses for the association of endometriosis and uterine fibroids with risk of mortality at any age (NHSII, 1989-2019).

Table S13. Sensitivity analyses for the association of endometriosis and uterine fibroids with risk of premature mortality by

excluding women who died within 5 years since the diagnosis of endometriosis or uterine fibroids (NHSII, 1989-2019).

Table S14. Sensitivity analyses for the association of endometriosis and uterine fibroids with risk of premature mortality, with adjustment for baseline medicine intake and behavioural factors (n=110,091; NHSII, 1989-2019).

Table S15. Sensitivity analyses for the association of endometriosis and uterine fibroids with risk of premature mortality, with additional adjustment for race/ethnicity (n=110,091; NHSII, 1989-2019).

Table S16. Sensitivity analyses for the association of endometriosis with the risk of premature mortality by including endometriosis cases both with and without laparoscopic confirmation (n=110,111; NHSII, 1989-2019).

Table S17. Sensitivity analyses for the association of uterine fibroids with the risk of premature mortality by including uterine fibroid cases both with and without ultrasound or hysterectomy confirmation (n=110,096; NHSII, 1989-2019).

Table S18. Robustness to unmeasured confounding (E-values) for assessing the associations between endometriosis and uterine fibroids with risk of all-cause and cause-specific premature mortality (n=110,091; NHSII, 1989-2019).

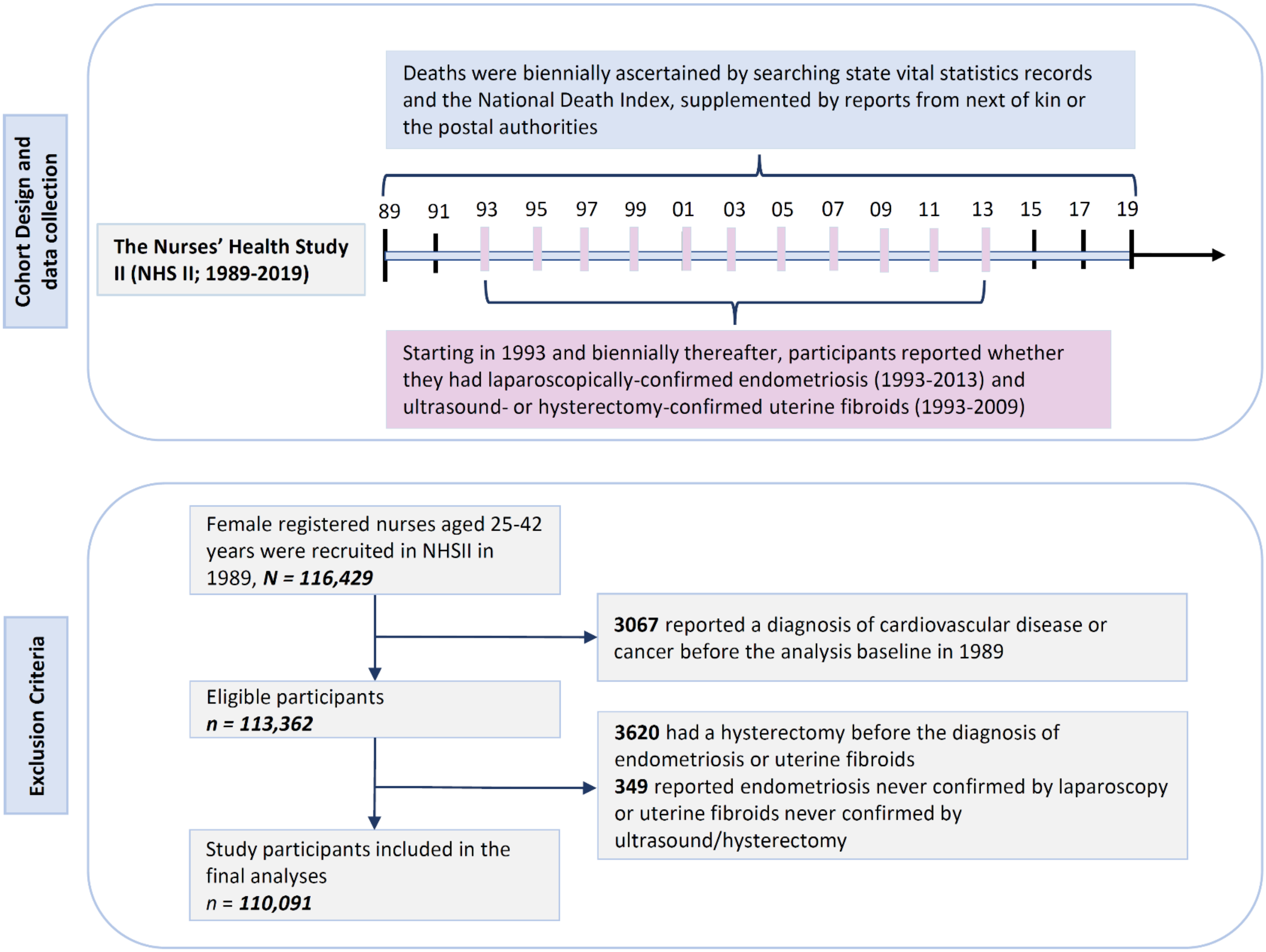

Figure S1. Study design and exclusion criteria

Table S1. Categories for causes of death.[a]		
**Causes of death**	**ICD-8 and 9 codes**	**Case numbers (n=4,356)**
Cancer	140-207	1,459
Breast cancer	174	476
Malignant neoplasm of digestive organs and peritoneum<br>150 Malignant neoplasm of oesophagus<br>151 Malignant neoplasm of stomach<br>152 Malignant neoplasm of small intestine, including duodenum<br>153 Malignant neoplasm of large intestine, except rectum<br>154 Malignant neoplasm of rectum and rectosigmoid junction<br>155 Malignant neoplasm of liver and intrahepatic bile ducts, specified as primary<br>156 Malignant neoplasm of gallbladder and bile ducts<br>157 Malignant neoplasm of pancreas<br>158 Malignant neoplasm of peritoneum and retroperitoneal tissue<br>159 Malignant neoplasm of unspecified digestive organs	150-159	256
Malignant neoplasm of the respiratory system<br>160 Malignant neoplasm of the nose, nasal cavities, middle ear, and accessory sinuses<br>161 Malignant neoplasm of larynx<br>162 Malignant neoplasm of trachea, bronchus, and lung<br>163 Malignant neoplasm of other and unspecified respiratory organs	160-163	193
Malignant neoplasm of gynaecological organs<br>180 Malignant neoplasm of cervix uteri<br>181 Chorionepithelioma<br>182 Other malignant neoplasms of the uterus<br>183 Malignant neoplasm of the ovary, Fallopian tube, and broad ligament<br>184 Malignant neoplasm of other and unspecified female gynaecological organs	180-184	127
Malignant neoplasm of urinary organs<br>185 Malignant neoplasm of prostate<br>188 Malignant neoplasm of bladder<br>189 Malignant neoplasm of other and unspecified urinary organs	185, 188-189	22
Neoplasms of lymphatic and haematopoietic tissue<br>200 Lymphosarcoma and reticulum-cell sarcoma<br>201 Hodgkin's disease<br>202 Other neoplasms of lymphoid tissue<br>203 Multiple myeloma<br>204 Lymphatic leukaemia<br>205 Myeloid leukaemia<br>206 Monocytic leukaemia<br>207 Other and unspecified leukaemia	200-207	148
Malignant neoplasm of bone, connective tissue, and skin<br>170 Malignant neoplasm of bone<br>171 Malignant neoplasm of connective and other soft tissue	170-174	65

172 Malignant melanoma of skin<br>173 Other malignant neoplasms of skin		
Malignant neoplasm of buccal cavity and pharynx<br>140 Malignant neoplasm of lip<br>141 Malignant neoplasm of tongue<br>142 Malignant neoplasm of salivary gland<br>143 Malignant neoplasm of gum<br>144 Malignant neoplasm of floor of mouth<br>145 Malignant neoplasm of other and unspecified parts of the mouth<br>146 Malignant neoplasm of oropharynx<br>147 Malignant neoplasm of nasopharynx<br>148 Malignant neoplasm of hypopharynx<br>149 Malignant neoplasm of the pharynx, unspecified	140-149	11
Malignant neoplasm of other and unspecified sites	190-199	161
Cardiovascular disease<br>390-392 Acute rheumatic fever<br>393-398 Chronic rheumatic heart disease<br>400-404 Hypertensive disease<br>410-414 Ischaemic heart disease<br>420-429 Other forms of heart disease<br>430-438 Cerebrovascular disease<br>440-448 Diseases of arteries, arterioles and capillaries<br>450-458 Diseases of veins and lymphatics, and other diseases of the circulatory system<br>795 Sudden death	390-458, 795	304
Other causes	390-458	2,503
External causes of injury and poisoning<br>800-807 Railway accidents<br>810-819 Motor vehicle traffic accidents<br>820-823 Motor vehicle non-traffic accidents<br>825-827 Other road vehicle accidents<br>830-838 Water transport accidents<br>840-845 Air and space transport accidents<br>850-859 Accidental poisoning by drugs and medicaments<br>860-869 Accidental poisoning by other solid and liquid substances<br>870-877 Accidental poisoning by gases and vapours<br>880-887 Accidental falls<br>890-899 Accidents caused by fires and flames<br>900-909 Accidents due to natural and environmental factors<br>910-929 Other accidents<br>930-936 Surgical and medical complications and misadventures<br>940-949 Late effects of accidental injury<br>960-969 Homicide and injury purposely inflicted by other persons	800-949, 960-999	264

970-978 Legal intervention<br>980-989 Injury undetermined whether accidentally or purposely inflicted<br>990-999 Injury resulting from operations of war		
Senility and ill-defined diseases<br>790 Nervousness and debility<br>791 Headache<br>792 Uraemia<br>793 Observation, without need for further medical care<br>794 Senility without mention of psychosis<br>796 Other ill-defined and unknown causes of morbidity and mortality	790-794, 796	175
Suicide<br>950 Suicide and self-inflicted poisoning by solid or liquid substances<br>951 Suicide and self-inflicted poisoning by gases in domestic use<br>952 Suicide and self-inflicted poisoning by other gases<br>953 Suicide and self-inflicted injury by hanging, strangulation, and suffocation<br>954 Suicide and self-inflicted injury by submersion (drowning)<br>955 Suicide and self-inflicted injury by firearms and explosives<br>956 Suicide and self-inflicted injury by cutting and piercing instruments<br>957 Suicide and self-inflicted injury by jumping from a high place<br>958 Suicide and self-inflicted injury by other and unspecified means<br>959 Late effect of self-inflicted injury	950-959	123
Respiratory disease<br>460-466 Acute respiratory infections (except influenza)<br>470-474 Influenza<br>480-486 Pneumonia<br>490-493 Bronchitis, emphysema and asthma<br>500-508 Other diseases of the upper respiratory tract<br>510-519 Other diseases of the respiratory system	460-519	90
Diseases of the nervous system and sense organs<br>320-324 Inflammatory diseases of central nervous system<br>330-333 Hereditary and familial diseases of the nervous system<br>340-349 Other diseases of the central nervous system<br>350-358 Diseases of nerves and peripheral ganglia<br>360-369 Inflammatory diseases of the eye<br>370-379 Other diseases and conditions of eyes<br>380-389 Diseases of the ear and mastoid process	320-389	86
Infectious and parasitic diseases<br>000-009 Intestinal infectious diseases<br>010-019 Tuberculosis<br>020-027 Zoonotic bacterial diseases<br>030-039 Other bacterial diseases<br>040-046 Poliomyelitis and other enterovirus diseases of the central nervous system	000-136	84

050-057 Viral diseases accompanied by exanthem<br>060-068 Arthropod-borne viral diseases<br>070-079 Other viral diseases<br>080-089 Rickettsioses and other arthropod-borne diseases<br>090-099 Syphilis and other venereal diseases<br>100-104 Other spirochaetal diseases<br>110-117 Mycoses<br>120-129 Helminthiases<br>130-136 Other infective and parasitic diseases		
Diseases of the digestive system<br>520-529 Diseases of the oral cavity, salivary glands and jaws<br>530-537 Diseases of the oesophagus, stomach, and duodenum<br>540-543 Appendicitis<br>544-544 Hernia of the abdominal cavity<br>560-569 Other diseases of the intestine and peritoneum<br>570-577 Diseases of the liver, gallbladder, and pancreas	520-577	83
Endocrine, nutritional, and metabolic diseases or immunity disorders<br>240-246 Diseases of the thyroid gland<br>250-258 Diseases of other endocrine glands<br>260-269 Avitaminoses and other nutritional deficiency<br>270-279 Other metabolic diseases	240-279	61
Mental disorders<br>290-299 Psychoses<br>300-309 Neuroses, personality disorders, and other nonpsychotic mental disorders<br>310-315 Mental retardation	290-315	45
Diseases of the musculoskeletal system and connective tissue<br>710-718 Arthritis and rheumatism, except rheumatic fever<br>720-729 Osteomyelitis and other diseases of bone and joint<br>730-738 Other diseases of the musculoskeletal system	710-738	34
Diseases of the genito-urinary system<br>580-584 Nephritis and nephrosis<br>590-599 Other diseases of the urinary system<br>600-607 Diseases of male genital organs<br>610-616 Diseases of the breast, ovary, Fallopian tube, and parametrium<br>620-629 Diseases of the uterus and other female gynaecological organs	580-629	26
Benign neoplasm	208-239	10
Congenital anomalies	740-759	8
Diseases of the blood and blood-forming organs	280-289	6
Complications of pregnancy, childbirth, and the puerperium	630-676, 760-779	5
Symptoms referable to systems or organs	780-789	3
Uncertain (uncertain cause of death)	-	1,490

[a]We classified participants according to the Statistics Netherlands' Database and U.S. Public Health Service and National Center for Health Statistics.

Text S1. The process of constructing a directed acyclic graph (DAG) and determining potential confounders

The DAG depicting covariates with different roles in the associations between endometriosis and uterine fibroids with risk of premature mortality was constructed by following the guidance from "Evidence Synthesis for Constructing Directed Acyclic Graphs" (ESC-DAGs).[1] First, a pool of covariates related to endometriosis, uterine fibroids, and/or mortality was determined by a series of systematic literature reviews. Second, a saturated DAG was assumed by drawing directed or undirected edges between all variables, including the exposure variable (endometriosis and uterine fibroids), the outcome variable (mortality), and the identified covariates, using DAGitty v3.1 (available at https://dagitty.net/dags.html). Third, each of the assumed causal relationships in the saturated DAG was assessed by sequential causal criteria (including temporality, validity, and theoretical support). Finally, we identified the following confounding factors existing in the Nurses' Health Study II: age,[2-4] history of infertility,[5,6] BMI,[2,3,7] menstrual cycle length,[2,8,9] age at menarche,[2,5,8] postmenopausal hormone therapy,[10-12] non-aspirin NSAID use,[10,13,14] aspirin use,[11,14,15] oral contraceptive use,[2,3,16] cigarette smoking status,[2,3,7] physical activity,[3,7,8] diet quality.[2,3,7,8] In the primary multivariable models, we adjusted for age, race/ethnicity, BMI at age 18 years, menstrual cycle length at age 18-22 years, age at menarche, postmenopausal hormone therapy, and time-varying non-aspirin NSAID use, aspirin use, and oral contraceptive use. In the final multivariable models, we further adjusted for time-varying BMI, smoking status, physical activity, and AHEI 2010 diet quality score, which could also serve as mediators.

References:

1. Ferguson KD, McCann M, Katikireddi SV, et al. Evidence synthesis for constructing directed acyclic graphs (ESC-DAGs): a novel and systematic method for building directed acyclic graphs. Int J Epidemiol 2020;49(1):322-329. DOI: 10.1093/ije/dyz150.
2. Vigano P, Parazzini F, Somigliana E, Vercellini P. Endometriosis: epidemiology and aetiological factors. Best Pract Res Clin Obstet Gynaecol 2004;18(2):177-200. DOI: 10.1016/j.bpobgyn.2004.01.007.
3. Pavone D, Clemenza S, Sorbi F, Fambrini M, Petraglia F. Epidemiology and Risk Factors of Uterine Fibroids. Best Pract Res Clin Obstet Gynaecol 2018;46:3-11. DOI: 10.1016/j.bpobgyn.2017.09.004.
4. Lozano R, Naghavi M, Foreman K, et al. Global and regional mortality from 235 causes of death for 20 age groups in 1990 and 2010: a systematic analysis for the Global Burden of Disease Study 2010. Lancet 2012;380(9859):2095-128. DOI: 10.1016/S0140-6736(12)61728-0.
5. de Ziegler D, Borghese B, Chapron C. Endometriosis and infertility: pathophysiology and management. Lancet 2010;376(9742):730-8. DOI: 10.1016/S0140-6736(10)60490-4.
6. Wang YX, Farland LV, Wang S, et al. Association of infertility with premature mortality among US women: Prospective cohort study. Lancet Reg Health Am 2022;7. DOI: 10.1016/j.lana.2021.100122.
7. van Dam RM, Li T, Spiegelman D, Franco OH, Hu FB. Combined impact of lifestyle factors on mortality: prospective cohort study in US women. BMJ 2008;337:a1440. DOI: 10.1136/bmj.a1440.
8. Shafrir AL, Farland LV, Shah DK, et al. Risk for and consequences of endometriosis: A critical epidemiologic review. Best Pract Res Clin Obstet Gynaecol 2018;51:1-15. DOI: 10.1016/j.bpobgyn.2018.06.001.
9. Wang YX, Arvizu M, Rich-Edwards JW, et al. Menstrual cycle regularity and length across the reproductive lifespan and risk of premature mortality: prospective cohort study. BMJ 2020;371:m3464. DOI: 10.1136/bmj.m3464.
10. Edi R, Cheng T. Endometriosis: Evaluation and Treatment. Am Fam Physician 2022;106(4):397-404. (https://www.ncbi.nlm.nih.gov/pubmed/36260896).
11. Gao M, Guo KM, Wei YM, et al. Aspirin inhibits the proliferation of human uterine leiomyoma cells by downregulation of K-Ras-p110alpha interaction. Oncol Rep 2017;38(4):2507-2517. DOI: 10.3892/or.2017.5915.
12. Mishra SR, Chung HF, Waller M, Mishra GD. Duration of estrogen exposure during reproductive years, age at menarche and age at menopause, and risk of cardiovascular disease events, all-cause and cardiovascular mortality: a systematic review and meta-analysis. BJOG 2021;128(5):809-821. DOI:

10.1111/1471-0528.16524.
13. Sinai Talaulikar V. Medical therapy for fibroids: An overview. Best Pract Res Clin Obstet Gynaecol 2018;46:48-56. DOI: 10.1016/j.bpobgyn.2017.09.007.
14. Hurwitz LM, Joshu CE, Barber JR, et al. Aspirin and Non-Aspirin NSAID Use and Prostate Cancer Incidence, Mortality, and Case Fatality in the Atherosclerosis Risk in Communities Study. Cancer Epidemiol Biomarkers Prev 2019;28(3):563-569. DOI: 10.1158/1055-9965.EPI-18-0965.
15. Wang L, Zhang J, Zhang H, et al. Low-dose aspirin can downregulate progesterone resistance and increase the expression of LIF in endometriosis during the implantation window. Gynecol Endocrinol 2021;37(8):725-729. DOI: 10.1080/09513590.2021.1918663.
16. Charlton BM, Rich-Edwards JW, Colditz GA, et al. Oral contraceptive use and mortality after 36 years of follow-up in the Nurses' Health Study: prospective cohort study. BMJ 2014;349:g6356. DOI: 10.1136/bmj.g6356.

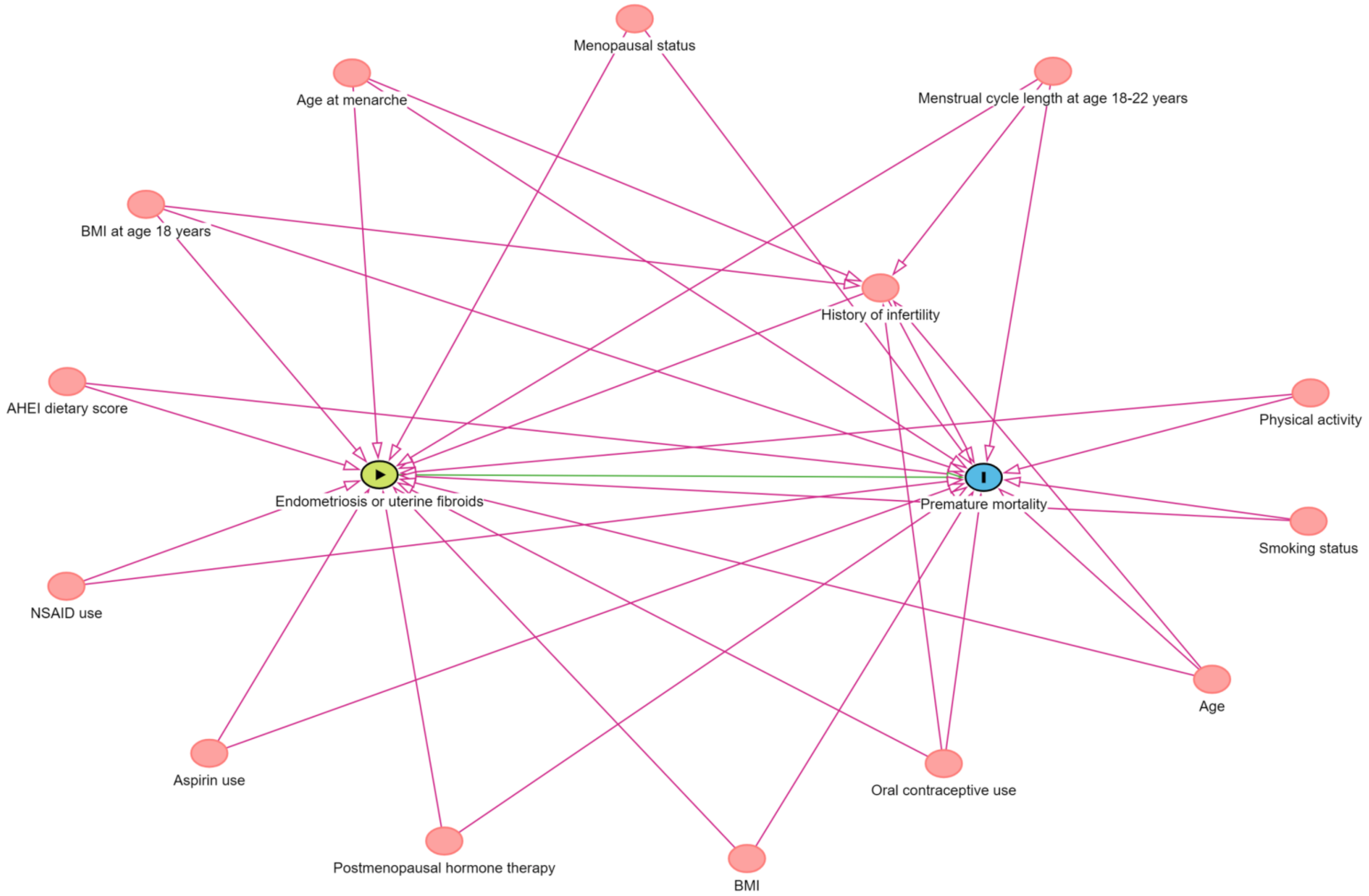

Figure S2. The final directed acyclic graph (DAG) depicting the assumed causal relationship between endometriosis and uterine fibroids with the risk of premature mortality, and their covariates. In the final DAG, each node (i.e. each circle) represents a specific variable and a directed edge (i.e. a line with an arrow) between a pair of nodes represents a causal relationship between them, starting from the cause and ending at the outcome. In the centre of the DAG figure, the green node of endometriosis and uterine fibroids is the exposure variable, the blue node of premature mortality is the outcome variable, and the green line between them represents the causal path of interest in this study.

Table S2. Hazard ratio (HR) (95% confidence interval (CI)) for the risk of all-cause and cause-specific premature mortality (before age 70 y) according to the joint occurrence of endometriosis and uterine fibroids (n=110,091; NHSII, 1989-2019).

Type of death	No	Laparoscopically confirmed endometriosis only	Ultrasound- or hysterectomy-confirmed uterine fibroids only	Both endometriosis and uterine fibroids
**All death**				
Events, No.	3,091	407	679	179
Crude incidence per 1000 person-years	1.33	1.92	1.83	2.23
Crude HR (95% CI)[a]	1.00 [Reference]	1.18 (1.07 to 1.31)	0.90 (0.83 to 0.98)	1.13 (0.97 to 1.32)
Adjusted HR (95% CI)[b]	1.00 [Reference]	1.32 (1.19 to 1.47)	1.02 (0.93 to 1.11)	1.31 (1.12 to 1.53)
**CVD**				
Events, No.	220	19	50	15
Crude incidence per 1000 person-years	0.09	0.09	0.13	0.19
Crude HR (95% CI)[a]	1.00 [Reference]	0.82 (0.51 to 1.31)	1.00 (0.73 to 1.37)	1.47 (0.87 to 2.49)
Adjusted HR (95% CI)[b]	1.00 [Reference]	0.93 (0.57 to 1.51)	1.07 (0.78 to 1.48)	1.61 (0.93 to 2.76)
**Cancer**				
Events, No.	1,020	128	262	49
Crude incidence per 1000 person-years	0.44	0.60	0.70	0.61
Crude HR (95% CI)[a]	1.00 [Reference]	1.18 (0.98 to 1.42)	1.12 (0.98 to 1.29)	1.01 (0.76 to 1.34)
Adjusted HR (95% CI)[b]	1.00 [Reference]	1.34 (1.10 to 1.62)	1.28 (1.11 to 1.47)	1.20 (0.90 to 1.61)
**Respiratory diseases**				
Events, No.	63	14	10	3
Crude incidence per 1000 person-years	0.03	0.07	0.03	0.04
Crude HR (95% CI)[a]	1.00 [Reference]	2.07 (1.16 to 3.70)	0.65 (0.33 to 1.27)	0.86 (0.27 to 2.76)
Adjusted HR (95% CI)[b]	1.00 [Reference]	2.21 (1.19 to 4.10)	0.72 (0.36 to 1.43)	1.00 (0.30 to 3.27)
**All other causes**				
Events, No.	1,788	246	357	112
Crude incidence per 1000 person-years	0.77	1.16	0.96	1.39
Crude HR (95% CI)[a]	1.00 [Reference]	1.20 (1.05 to 1.37)	0.78 (0.70 to 0.88)	1.17 (0.96 to 1.41)
Adjusted HR (95% CI)[b]	1.00 [Reference]	1.31 (1.14 to 1.51)	0.88 (0.79 to 0.99)	1.34 (1.10 to 1.63)

[a]The analyses were stratified jointly by participants' own age in months at the start of follow-up and calendar years of the current questionnaire cycle. [b]Models were additionally adjusted for history of infertility (yes, no [reference]), BMI at age 18 years (<18.5, 18.5-24.9 [reference], 25-29.9, 20-34.9, ≥35 kg/m^2), menstrual cycle length at age 18-22 years (<26, 26-31 [reference], 32-50, or ≥50 days or too irregular to estimate), age at menarche (<12 [reference], 12, 13, or ≥14 years of age), as well as time-varying non-aspirin NSAID use (yes, no [reference]), aspirin use (yes, no [reference]), oral contraceptive use (current/former, no [reference]), postmenopausal hormone therapy (never [reference], past, current), BMI (<24.9 [reference], 25-29.9, 30-34.9, or ≥35 kg/m^2), smoking status (never [reference], former, current 1-34 cigarettes/day, or current ≥35 cigarettes/day), physical activity (0 [reference]), 0.1-1.0, 1.1-2.4, 2.5-5.9, or ≥6 h/week), and Alternative Healthy Eating Index 2010 diet quality scores (quintiles, with the lowest quintile [reference] representing the least healthy diet).

Table S3. Hazard ratio (HR) (95% confidence interval (CI)) for the risk of premature cancer mortality according to confirmed endometriosis and uterine fibroids diagnosis, stratified by behavioural and reproductive factors (n=110,091; NHSII, 1989-2019).[a]

**Stratified factors**	Laparoscopically- confirmed endometriosis		Ultrasound- or hysterectomy-confirmed uterine fibroids	
	**No**	**Yes**	**No**	**Yes**
**Race/ethnicity**				
Non-Hispanic White (n=1,311 deaths)	1.00 [Reference]	1.27 (1.07 to 1.51)	1.00 [Reference]	1.26 (1.10 to 1.44)
Other (n=148 deaths)	1.00 [Reference]	0.71 (0.35 to 1.43)	1.00 [Reference]	0.86 (0.54 to 1.36)
P for interaction[b]	0.63		0.22	
**Diet quality[a]**				
Top 40% (n=464 deaths)	1.00 [Reference]	1.13 (0.84 to 1.52)	1.00 [Reference]	1.20 (0.96 to 1.49)
Bottom 60% (n=995 deaths)	1.00 [Reference]	1.25 (1.02 to 1.52)	1.00 [Reference]	1.23 (1.05 to 1.44)
P for interaction[b]	0.66		0.91	
**Smoking status**				
Never smokers (n=806 deaths)	1.00 [Reference]	1.16 (0.92 to 1.46)	1.00 [Reference]	1.18 (0.99 to 1.41)
Current/former smokers (n=653 deaths)	1.00 [Reference]	1.34 (1.06 to 1.70)	1.00 [Reference]	1.26 (1.04 to 1.53)
P for interaction[b]	0.63		0.91	
**BMI**				
<25 kg/m^2 (n=681 deaths)	1.00 [Reference]	1.29 (1.01 to 1.64)	1.00 [Reference]	1.25 (1.03 to 1.53)
≥25 kg/m^2 (n=778 deaths)	1.00 [Reference]	1.15 (0.92 to 1.44)	1.00 [Reference]	1.18 (0.99 to 1.40)
P for interaction[b]	0.63		0.79	
**Physical activity**				
≥30 min/day (n=488 deaths)	1.00 [Reference]	1.52 (1.14 to 2.02)	1.00 [Reference]	1.13 (0.89 to 1.43)
<30 min/day (n=971 deaths)	1.00 [Reference]	1.10 (0.90 to 1.35)	1.00 [Reference]	1.25 (1.07 to 1.46)
P for interaction[b]	0.63		0.44	
**Postmenopausal hormone therapy**				
Yes (n=636 deaths)	1.00 [Reference]	1.10 (0.90 to 1.35)	1.00 [Reference]	1.12 (0.95 to 1.33)
No (n=823 deaths)	1.00 [Reference]	1.23 (0.92 to 1.64)	1.00 [Reference]	1.20 (0.98 to 1.47)
P for interaction[b]	0.79		0.91	
**Parity**				
Nulliparous (n=319 deaths)	1.00 [Reference]	0.92 (0.65 to 1.29)	1.00 [Reference]	1.80 (1.39 to 2.33)
Parous (n=1,140 deaths)	1.00 [Reference]	1.34 (1.11 to 1.62)	1.00 [Reference]	1.09 (0.93 to 1.26)
P for interaction[b]	0.63		0.06	
**History of infertility**				
No (n=1,075 deaths)	1.00 [Reference]	1.17 (0.93 to 1.47)	1.00 [Reference]	1.21 (1.03 to 1.41)
Yes (n=384 deaths)	1.00 [Reference]	1.30 (1.02 to 1.66)	1.00 [Reference]	1.24 (0.97 to 1.57)
P for interaction[b]	0.81		0.95	
**History of hysterectomy**				
No (n=1,220 deaths)	1.00 [Reference]	0.96 (0.75 to 1.24)	1.00 [Reference]	1.16 (0.98 to 1.38)
Yes (n=239 deaths)	1.00 [Reference]	1.14 (0.88 to 1.48)	1.00 [Reference]	0.76 (0.58 to 1.00)
P for interaction[b]	0.63		0.08	
**History of oophorectomy**				
No (n=1,162 deaths)	1.00 [Reference]	1.00 (0.78 to 1.29)	1.00 [Reference]	1.02 (0.86 to 1.21)
Yes (n=297 deaths)	1.00 [Reference]	0.88 (0.69 to 1.12)	1.00 [Reference]	1.06 (0.83 to 1.34)
P for interaction[b]	0.63		0.91	
**Asprin use**				
Never (n=1,181 deaths)	1.00 [Reference]	1.15 (0.95 to 1.40)	1.00 [Reference]	1.16 (1.00 to 1.35)
Current/former (n=278 deaths)	1.00 [Reference]	1.48 (1.07 to 2.03)	1.00 [Reference]	1.47 (1.12 to 1.91)
P for interaction[b]	0.63		0.70	

**Non-aspirin NSAID use**				
Never (n=967 deaths)	1.00 [Reference]	1.21 (0.98 to 1.49)	1.00 [Reference]	1.14 (0.97 to 1.35)
Current/former (n=492 deaths)	1.00 [Reference]	1.26 (0.98 to 1.64)	1.00 [Reference]	1.33 (1.08 to 1.64)
P for interaction[b]	0.93		0.79	
**Spontaneous abortion history**				
No (n=1,153 deaths)	1.00 [Reference]	1.25 (1.03 to 1.50)	1.00 [Reference]	1.16 (1.00 to 1.35)
Yes (n=306 deaths)	1.00 [Reference]	1.15 (0.82 to 1.61)	1.00 [Reference]	1.42 (1.09 to 1.84)
P for interaction[b]	0.86		0.50	
**Long or irregular menstrual cycles in adulthood**				
No (n=1,241 deaths)	1.00 [Reference]	1.29 (1.08 to 1.53)	1.00 [Reference]	1.22 (1.06 to 1.40)
Yes (n=218 deaths)	1.00 [Reference]	0.98 (0.61 to 1.55)	1.00 [Reference]	1.24 (0.89 to 1.73)
P for interaction[b]	0.63		0.91	
**Oral contraceptive use**				
Never (n=399 deaths)	1.00 [Reference]	1.04 (0.67 to 1.60)	1.00 [Reference]	1.06 (0.78 to 1.45)
Current/former (n=1,060 deaths)	1.00 [Reference]	1.27 (1.07 to 1.52)	1.00 [Reference]	1.25 (1.08 to 1.44)
P for interaction[b]	0.63		0.23	

[a] Models were adjusted for age (continuous), history of infertility (yes, no [reference]), BMI at age 18 years (<18.5, 18.5-24.9 [reference], 25-29.9, 20-34.9, ≥35 kg/m^2), menstrual cycle length at age 18-22 years (<26, 26-31 [reference], 32-50, or ≥50 days or too irregular to estimate), age at menarche (<12 [reference], 12, 13, or ≥14 years of age), as well as time-varying non-aspirin NSAID use (yes, no [reference]), aspirin use (yes, no [reference]), oral contraceptive use (current/former, no [reference]), postmenopausal hormone therapy (never [reference], past, current), BMI (<24.9 [reference], 25-29.9, 30-34.9, or ≥35 kg/m^2), smoking status (never [reference], former, current 1-34 cigarettes/day, or current ≥35 cigarettes/day), physical activity (0 [reference]), 0.1-1.0, 1.1-2.4, 2.5-5.9, or ≥6 h/week), and Alternative Healthy Eating Index 2010 diet quality scores (quintiles, with the lowest quintile [reference] representing the least healthy diet), excluding the stratifying variable. [b]FDR-adjusted P-values.

Table S4. Hazard ratio (HR) (95% confidence interval (CI)) for the risk of cause-specific mortality in relation to laparoscopically confirmed endometriosis based on competing-risk regression models (n=110,091; NHSII, 1989-2019).

**Causes of mortality**	**No endometriosis**	**Laparoscopically confirmed endometriosis**
Cancer		
Death cases	1,282	177
Crude incidence per 1000 person-years	0.47	0.61
Age-adjusted models[a]	1.00 [Reference]	1.10 (0.94 to 1.29)
Multivariable model 1[b]	1.00 [Reference]	1.27 (1.08 to 1.49)
Multivariable model 2[c]	1.00 [Reference]	1.23 (1.05 to 1.44)
CVD		
Death cases	270	34
Crude incidence per 1000 person-years	0.10	0.12
Age-adjusted models[a]	1.00 [Reference]	1.03 (0.72 to 1.47)
Multivariable model 1[b]	1.00 [Reference]	1.19 (0.83 to 1.71)
Multivariable model 2[c]	1.00 [Reference]	1.15 (0.81 to 1.65)
Respiratory diseases		
Death cases	73	17
Crude incidence per 1000 person-years	0.03	0.06
Age-adjusted models[a]	1.00 [Reference]	1.82 (1.07 to 3.08)
Multivariable model 1[b]	1.00 [Reference]	2.09 (1.23 to 3.55)
Multivariable model 2[c]	1.00 [Reference]	2.02 (1.19 to 3.43)
All other deaths		
Death cases	2,145	358
Crude incidence per 1000 person-years	0.79	1.23
Age-adjusted models[a]	1.00 [Reference]	1.25 (1.11 to 1.39)
Multivariable model 1[b]	1.00 [Reference]	1.40 (1.24 to 1.57)
Multivariable model 2[c]	1.00 [Reference]	1.35 (1.20 to 1.52)
P for heterogeneity[d]		
Age-adjusted models[a]	0.21	-
Multivariable model 1[b]	0.29	-
Multivariable model 2[c]	0.29	-

[a]In age-adjusted Cox proportional hazard regression models, the analyses were stratified jointly by participants' own age in months at the start of follow-up and calendar years of the current questionnaire cycle. [b]Multivariable models were further adjusted for history of infertility (yes, no [reference]), BMI at age 18 years (<18.5, 18.5-24.9 [reference], 25-29.9, 20-34.9, ≥35 kg/m^2), menstrual cycle length at age 18-22 years (<26, 26-31 [reference], 32-50, or ≥50 days or too irregular to estimate), age at menarche (<12 [reference], 12, 13, or ≥14 years of age), as well as time-varying postmenopausal hormone therapy (never [reference], past, current), non-aspirin NSAID use (yes, no [reference]), aspirin use (yes, no [reference]), and oral contraceptive use (current/former, no [reference]). [c]Full models were further adjusted for time-varying BMI (<24.9 [reference], 25-29.9, 30-34.9, or ≥35 kg/m^2), cigarette smoking status (never [reference], former, current 1-34 cigarettes/day, or current ≥35 cigarettes/day), physical activity (0 [reference]), 0.1-1.0, 1.1-2.4, 2.5-5.9, or ≥6 h/week), and Alternative Healthy Eating Index 2010 diet quality scores (quintiles, with the lowest quintile [reference] representing the least healthy diet). [d]P for heterogeneity of the association of endometriosis and different types of mortality were tested using the Wald test.

Table S5. Hazard ratio (HR) (95% confidence interval (CI)) for the risk of cause-specific mortality in relation to uterine fibroids based on competing-risk regression models (n=110,091; NHSII, 1989-2019).

**Causes of mortality**	**No uterine fibroids**	**Ultrasound- or hysterectomy-confirmed uterine fibroids**
Cancer		
Death cases	1,148	311
Crude incidence per 1000 person-years	0.45	0.69
Age-adjusted models[a]	1.00 [Reference]	1.09 (0.96 to 1.24)
Multivariable model 1[b]	1.00 [Reference]	1.21 (1.06 to 1.37)
Multivariable model 2[c]	1.00 [Reference]	1.23 (1.08 to 1.39)
CVD		
Death cases	239	65
Crude incidence per 1000 person-years	0.09	0.14
Age-adjusted models[a]	1.00 [Reference]	1.12 (0.85 to 1.48)
Multivariable model 1[b]	1.00 [Reference]	1.24 (0.94 to 1.64)
Multivariable model 2[c]	1.00 [Reference]	1.25 (0.95 to 1.66)
Respiratory diseases		
Death cases	77	13
Crude incidence per 1000 person-years	0.03	0.03
Age-adjusted models[a]	1.00 [Reference]	0.64 (0.35 to 1.16)
Multivariable model 1[b]	1.00 [Reference]	0.70 (0.39 to 1.27)
Multivariable model 2[c]	1.00 [Reference]	0.71 (0.39 to 1.29)
All other deaths		
Death cases	2,034	469
Crude incidence per 1000 person-years	0.80	1.04
Age-adjusted models[a]	1.00 [Reference]	0.84 (0.76 to 0.93)
Multivariable model 1[b]	1.00 [Reference]	0.91 (0.82 to 1.01)
Multivariable model 2[c]	1.00 [Reference]	0.92 (0.83 to 1.02)
P for heterogeneity[d]		
Age-adjusted models[a]	0.004	-
Multivariable model 1[b]	0.002	-
Multivariable model 2[c]	0.002	-

[a]In age-adjusted Cox proportional hazard regression models, the analyses were stratified jointly by participants' own age in months at the start of follow-up and calendar years of the current questionnaire cycle. [b]Multivariable models were further adjusted for history of infertility (yes, no [reference]), BMI at age 18 years (<18.5, 18.5-24.9 [reference], 25-29.9, 20-34.9, ≥35 kg/m^2), menstrual cycle length at age 18-22 years (<26, 26-31 [reference], 32-50, or ≥50 days or too irregular to estimate), age at menarche (<12 [reference], 12, 13, or ≥14 years of age), as well as time-varying postmenopausal hormone therapy (never [reference], past, current), non-aspirin NSAID use (yes, no [reference]), aspirin use (yes, no [reference]), and oral contraceptive use (current/former, no [reference]). [c]Full models were further adjusted for time-varying BMI (<24.9 [reference], 25-29.9, 30-34.9, or ≥35 kg/m^2), cigarette smoking status (never [reference], former, current 1-34 cigarettes/day, or current ≥35 cigarettes/day), physical activity (0 [reference]), 0.1-1.0, 1.1-2.4, 2.5-5.9, or ≥6 h/week), and Alternative Healthy Eating Index 2010 diet quality scores (quintiles, with the lowest quintile [reference] representing the least healthy diet). [d]P for heterogeneity of the association of uterine fibroids and different types of mortality were tested using the Wald test.

Table S6. Sensitivity analyses for the association of endometriosis and uterine fibroids with the risk of premature mortality by excluding women who never returned follow-up questionnaires (N=108,820; NHSII, 1989-2019).

Cause-specific mortality	Laparoscopically confirmed endometriosis		Ultrasound- or hysterectomy-confirmed uterine fibroids	
	No	Yes	No	Yes
**All death**				
Events, No.	3,619	586	3,347	858
Crude incidence per 1000 person-years	1.36	2.01	1.34	1.90
HR (95% CI) [a]	1.00 [Reference]	1.32 (1.20 to 1.45)	1.00 [Reference]	1.03 (0.95 to 1.11)
**CVD**				
Events, No.	259	34	228	65
Crude incidence per 1000 person-years	0.10	0.12	0.09	0.14
HR (95% CI) [a]	1.00 [Reference]	1.11 (0.76 to 1.62)	1.00 [Reference]	1.18 (0.88 to 1.57)
**Cancer**				
Events, No.	1,240	177	1,106	311
Crude incidence per 1000 person-years	0.46	0.61	0.44	0.69
HR (95% CI) [a]	1.00 [Reference]	1.23 (1.04 to 1.45)	1.00 [Reference]	1.22 (1.07 to 1.39)
**Respiratory diseases**				
Events, No.	69	17	73	13
Crude incidence per 1000 person-years	0.03	0.06	0.03	0.03
HR (95% CI) [a]	1.00 [Reference]	2.00 (1.14 to 3.52)	1.00 [Reference]	0.67 (0.36 to 1.23)
**All other causes**				
Events, No.	2,051	358	1,940	469
Crude incidence per 1000 person-years	0.77	1.23	0.77	1.04
HR (95% CI) [a]	1.00 [Reference]	1.37 (1.22 to 1.54)	1.00 [Reference]	0.92 (0.83 to 1.02)

[a] Models were adjusted for age (continuous), history of infertility (yes, no [reference]), BMI at age 18 years (<18.5, 18.5-24.9 [reference], 25-29.9, 20-34.9, ≥35 kg/m^2), menstrual cycle length at age 18-22 years (<26, 26-31 [reference], 32-50, or ≥50 days or too irregular to estimate), age at menarche (<12 [reference], 12, 13, or ≥14 years of age), as well as time-varying non-aspirin NSAID use (yes, no [reference]), aspirin use (yes, no [reference]), oral contraceptive use (current/former, no [reference]), postmenopausal hormone therapy (never [reference], past, current), BMI (<24.9 [reference], 25-29.9, 30-34.9, or ≥35 kg/m^2), smoking status (never [reference], former, current 1-34 cigarettes/day, or current ≥35 cigarettes/day), physical activity (0 [reference]), 0.1-1.0, 1.1-2.4, 2.5-5.9, or ≥6 h/week), and Alternative Healthy Eating Index 2010 diet quality scores (quintiles, with the lowest quintile [reference] representing the least healthy diet).

Table S7. Sensitivity analyses for the association of endometriosis and uterine fibroids with the risk of premature mortality using the Markov chain Monte Carlo method of multiple imputations procedure to replace covariates with missing values (n=110,091; NHSII, 1989-2019).

Cause-specific mortality	Laparoscopically confirmed endometriosis		Ultrasound- or hysterectomy-confirmed uterine fibroids	
	No	Yes	No	Yes
**All death**				
Events, No.	3,770	586	3,498	858
Crude incidence per 1000 person-years	1.40	2.01	1.38	1.90
HR (95% CI) [a]	1.00 [Reference]	1.32 (1.20 to 1.44)	1.00 [Reference]	1.01 (0.93 to 1.09)
**CVD**				
Events, No.	270	34	239	65
Crude incidence per 1000 person-years	0.10	0.12	0.09	0.14
HR (95% CI) [a]	1.00 [Reference]	1.13 (0.78 to 1.64)	1.00 [Reference]	1.14 (0.86 to 1.52)
**Cancer**				
Events, No.	1,282	177	1,148	311
Crude incidence per 1000 person-years	0.47	0.61	0.45	0.69
HR (95% CI) [a]	1.00 [Reference]	1.22 (1.04 to 1.44)	1.00 [Reference]	1.22 (1.07 to 1.38)
**Respiratory diseases**				
Events, No.	73	17	77	13
Crude incidence per 1000 person-years	0.03	0.06	0.03	0.03
HR (95% CI) [a]	1.00 [Reference]	1.94 (1.11 to 3.40)	1.00 [Reference]	0.64 (0.35 to 1.18)
**All other causes**				
Events, No.	2,145	358	2,034	469
Crude incidence per 1000 person-years	0.79	1.23	0.80	1.04
HR (95% CI) [a]	1.00 [Reference]	1.37 (1.21 to 1.54)	1.00 [Reference]	0.90 (0.81 to 1.00)

[a] Models were adjusted for age (continuous), history of infertility (yes, no [reference]), BMI at age 18 years (<18.5, 18.5-24.9 [reference], 25-29.9, 20-34.9, ≥35 kg/m^2), menstrual cycle length at age 18-22 years (<26, 26-31 [reference], 32-50, or ≥50 days or too irregular to estimate), age at menarche (<12 [reference], 12, 13, or ≥14 years of age), as well as time-varying non-aspirin NSAID use (yes, no [reference]), aspirin use (yes, no [reference]), oral contraceptive use (current/former, no [reference]), postmenopausal hormone therapy (never [reference], past, current), BMI (<24.9 [reference], 25-29.9, 30-34.9, or ≥35 kg/m^2), smoking status (never [reference], former, current 1-34 cigarettes/day, or current ≥35 cigarettes/day), physical activity (0 [reference]), 0.1-1.0, 1.1-2.4, 2.5-5.9, or ≥6 h/week), and Alternative Healthy Eating Index 2010 diet quality scores (quintiles, with the lowest quintile [reference] representing the least healthy diet).

Table S8. Sensitivity analyses for the association of endometriosis and uterine fibroids with the risk of premature mortality, with additional adjustment for night shift work (n=110,091; NHSII, 1989-2019).

Cause-specific mortality	Laparoscopically confirmed endometriosis		Ultrasound- or hysterectomy-confirmed uterine fibroids	
	No	Yes	No	Yes
**All death**				
Events, No.	3,770	586	3,498	858
Crude incidence per 1000 person-years	1.40	2.01	1.38	1.90
HR (95% CI) [a]	1.00 [Reference]	1.31 (1.19 to 1.43)	1.00 [Reference]	1.03 (0.95 to 1.11)
**CVD**				
Events, No.	270	34	239	65
Crude incidence per 1000 person-years	0.10	0.12	0.09	0.14
HR (95% CI) [a]	1.00 [Reference]	1.13 (0.77 to 1.64)	1.00 [Reference]	1.16 (0.87 to 1.55)
**Cancer**				
Events, No.	1,282	177	1,148	311
Crude incidence per 1000 person-years	0.47	0.61	0.45	0.69
HR (95% CI) [a]	1.00 [Reference]	1.22 (1.04 to 1.44)	1.00 [Reference]	1.22 (1.07 to 1.39)
**Respiratory diseases**				
Events, No.	73	17	77	13
Crude incidence per 1000 person-years	0.03	0.06	0.03	0.03
HR (95% CI) [a]	1.00 [Reference]	1.95 (1.11 to 3.41)	1.00 [Reference]	0.67 (0.36 to 1.23)
**All other causes**				
Events, No.	2,145	358	2,034	469
Crude incidence per 1000 person-years	0.79	1.23	0.80	1.04
HR (95% CI) [a]	1.00 [Reference]	1.35 (1.20 to 1.52)	1.00 [Reference]	0.92 (0.83 to 1.02)

[a] Models were adjusted for age (continuous), history of infertility (yes, no [reference]), BMI at age 18 years (<18.5, 18.5-24.9 [reference], 25-29.9, 20-34.9, ≥35 kg/m^2), menstrual cycle length at age 18-22 years (<26, 26-31 [reference], 32-50, or ≥50 days or too irregular to estimate), age at menarche (<12 [reference], 12, 13, or ≥14 years of age), as well as time-varying non-aspirin NSAID use (yes, no [reference]), aspirin use (yes, no [reference]), oral contraceptive use (current/former, no [reference]), postmenopausal hormone therapy (never [reference], past, current), BMI (<24.9 [reference], 25-29.9, 30-34.9, or ≥35 kg/m2), smoking status (never [reference], former, current 1-34 cigarettes/day, or current ≥35 cigarettes/day), physical activity (0 [reference]), 0.1-1.0, 1.1-2.4, 2.5-5.9, or ≥6 h/week), Alternative Healthy Eating Index 2010 diet quality scores (quintiles, with the lowest quintile [reference] representing the least healthy diet), and duration of rotating night shift work (never, 1-2, 3-5, 6-9, 10-14, 15-19, and ≥20 years).

Table S9. Sensitivity analyses for the association of endometriosis with the risk of premature mortality by excluding women from the comparison group who had uterine fibroids (n=106,096; NHSII, 1989-2019).

Cause-specific mortality	Laparoscopically confirmed endometriosis	
	No	Yes
**All death**		
Events, No.	3,091	586
Crude incidence per 1000 person-years	1.33	2.01
HR (95% CI) [a]	1.00 [Reference]	1.32 (1.20 to 1.45)
**CVD**		
Events, No.	220	34
Crude incidence per 1000 person-years	0.09	0.12
HR (95% CI) [a]	1.00 [Reference]	1.14 (0.77 to 1.67)
**Cancer**		
Events, No.	1,020	177
Crude incidence per 1000 person-years	0.44	0.61
HR (95% CI) [a]	1.00 [Reference]	1.32 (1.11 to 1.56)
**Respiratory diseases**		
Events, No.	63	17
Crude incidence per 1000 person-years	0.03	0.06
HR (95% CI) [a]	1.00 [Reference]	1.84 (1.03 to 3.28)
**All other causes**		
Events, No.	1,788	358
Crude incidence per 1000 person-years	0.77	1.23
HR (95% CI) [a]	1.00 [Reference]	1.31 (1.16 to 1.48)

[a] Models were adjusted for age (continuous), history of infertility (yes, no [reference]), BMI at age 18 years (<18.5, 18.5-24.9 [reference], 25-29.9, 20-34.9, ≥35 kg/m^2), menstrual cycle length at age 18-22 years (<26, 26-31 [reference], 32-50, or ≥50 days or too irregular to estimate), age at menarche (<12 [reference], 12, 13, or ≥14 years of age), as well as time-varying non-aspirin NSAID use (yes, no [reference]), aspirin use (yes, no [reference]), oral contraceptive use (current/former, no [reference]), postmenopausal hormone therapy (never [reference], past, current), BMI (<24.9 [reference], 25-29.9, 30-34.9, or ≥35 kg/m^2), smoking status (never [reference], former, current 1-34 cigarettes/day, or current ≥35 cigarettes/day), physical activity (0 [reference]), 0.1-1.0, 1.1-2.4, 2.5-5.9, or ≥6 h/week), and Alternative Healthy Eating Index 2010 diet quality scores (quintiles, with the lowest quintile [reference] representing the least healthy diet).

Table S10. Sensitivity analyses for the association of uterine fibroids with the risk of premature mortality by excluding women from the comparison group who had endometriosis (n=106,091; NHSII, 1989-2019).

Cause-specific mortality	Ultrasound- or hysterectomy- confirmed uterine fibroids	
	No	Yes
**All death**		
Events, No.	3,091	858
Crude incidence per 1000 person-years	1.33	1.90
HR (95% CI) [a]	1.00 [Reference]	1.07(0.99 to 1.16)
**CVD**		
Events, No.	220	65
Crude incidence per 1000 person-years	0.09	0.14
HR (95% CI) [a]	1.00 [Reference]	1.18 (0.88 to 1.58)
**Cancer**		
Events, No.	1,020	311
Crude incidence per 1000 person-years	0.44	0.69
HR (95% CI) [a]	1.00 [Reference]	1.26 (1.10 to 1.44)
**Respiratory diseases**		
Events, No.	63	13
Crude incidence per 1000 person-years	0.03	0.03
HR (95% CI) [a]	1.00 [Reference]	0.85 (0.45 to 1.58)
**All other causes**		
Events, No.	1,788	469
Crude incidence per 1000 person-years	0.77	1.04
HR (95% CI) [a]	1.00 [Reference]	0.96 (0.87 to 1.07)

[a] Models were adjusted for age (continuous), history of infertility (yes, no [reference]), BMI at age 18 years (<18.5, 18.5-24.9 [reference], 25-29.9, 20-34.9, ≥35 kg/m^2), menstrual cycle length at age 18-22 years (<26, 26-31 [reference], 32-50, or ≥50 days or too irregular to estimate), age at menarche (<12 [reference], 12, 13, or ≥14 years of age), as well as time-varying non-aspirin NSAID use (yes, no [reference]), aspirin use (yes, no [reference]), oral contraceptive use (current/former, no [reference]), postmenopausal hormone therapy (never [reference], past, current), BMI (<24.9 [reference], 25-29.9, 30-34.9, or ≥35 kg/m^2), smoking status (never [reference], former, current 1-34 cigarettes/day, or current ≥35 cigarettes/day), physical activity (0 [reference]), 0.1-1.0, 1.1-2.4, 2.5-5.9, or ≥6 h/week), and Alternative Healthy Eating Index 2010 diet quality scores (quintiles, with the lowest quintile [reference] representing the least healthy diet).

Table S11. Sensitivity analyses for the association of endometriosis and uterine fibroids with risk of premature mortality by redefined premature mortality as deaths before 65 years of age (NHSII, 1989-2019).

Cause-specific mortality	Laparoscopically confirmed endometriosis		Ultrasound- or hysterectomy- confirmed uterine fibroids	
	No	Yes	No	Yes
**All death**				
Events, No.	3,164	482	2,951	695
Crude incidence per 1000 person-years	1.17	1.65	1.16	1.54
HR (95% CI) [a]	1.00 [Reference]	1.33 (1.20 to 1.47)	1.00 [Reference]	1.07(0.98 to 1.16)
**CVD**				
Events, No.	254	33	227	60
Crude incidence per 1000 person-years	0.09	0.11	0.09	0.13
HR (95% CI) [a]	1.00 [Reference]	1.20 (0.82 to 1.75)	1.00 [Reference]	1.18 (0.88 to 1.59)
**Cancer**				
Events, No.	1,213	160	1,093	280
Crude incidence per 1000 person-years	0.45	0.55	0.43	0.62
HR (95% CI) [a]	1.00 [Reference]	1.19 (1.00 to 1.41)	1.00 [Reference]	1.19 (1.04 to 1.37)
**Respiratory diseases**				
Events, No.	69	13	71	11
Crude incidence per 1000 person-years	0.03	0.04	0.03	0.02
HR (95% CI) [a]	1.00 [Reference]	1.57 (0.84 to 2.94)	1.00 [Reference]	0.65 (0.34 to 1.24)
**All other causes**				
Events, No.	1,628	276	1,560	344
Crude incidence per 1000 person-years	0.60	0.94	0.61	0.76
HR (95% CI) [a]	1.00 [Reference]	1.44 (1.26 to 1.65)	1.00 [Reference]	0.99 (0.88 to 1.12)

[a] Models were adjusted for age (continuous), history of infertility (yes, no [reference]), BMI at age 18 years (<18.5, 18.5-24.9 [reference], 25-29.9, 20-34.9, ≥35 kg/m^2), menstrual cycle length at age 18-22 years (<26, 26-31 [reference], 32-50, or ≥50 days or too irregular to estimate), age at menarche (<12 [reference], 12, 13, or ≥14 years of age), as well as time-varying non-aspirin NSAID use (yes, no [reference]), aspirin use (yes, no [reference]), oral contraceptive use (current/former, no [reference]), postmenopausal hormone therapy (never [reference], past, current), BMI (<24.9 [reference], 25-29.9, 30-34.9, or ≥35 kg/m^2), smoking status (never [reference], former, current 1-34 cigarettes/day, or current ≥35 cigarettes/day), physical activity (0 [reference]), 0.1-1.0, 1.1-2.4, 2.5-5.9, or ≥6 h/week), and Alternative Healthy Eating Index 2010 diet quality scores (quintiles, with the lowest quintile [reference] representing the least healthy diet).

Table S12. Sensitivity analyses for the association of endometriosis and uterine fibroids with risk of mortality at any age (NHSII, 1989-2019).

Cause-specific mortality	Laparoscopically confirmed endometriosis		Ultrasound- or hysterectomy-confirmed uterine fibroids	
	No	Yes	No	Yes
**All death**				
Events, No.	3,871	609	3,588	892
Crude incidence per 1000 person-years	1.43	2.09	1.41	1.97
HR (95% CI) [a]	1.00 [Reference]	1.33 (1.21 to 1.45)	1.00 [Reference]	1.02 (0.95 to 1.11)
**CVD**				
Events, No.	273	34	241	66
Crude incidence per 1000 person-years	0.10	0.12	0.09	0.15
HR (95% CI) [a]	1.00 [Reference]	1.10 (0.76 to 1.60)	1.00 [Reference]	1.17 (0.88 to 1.55)
**Cancer**				
Events, No.	1,288	178	1,152	314
Crude incidence per 1000 person-years	0.48	0.61	0.45	0.69
HR (95% CI) [a]	1.00 [Reference]	1.22 (1.04 to 1.44)	1.00 [Reference]	1.22 (1.07 to 1.39)
**Respiratory diseases**				
Events, No.	74	17	78	13
Crude incidence per 1000 person-years	0.03	0.06	0.03	0.03
HR (95% CI) [a]	1.00 [Reference]	1.92 (1.10 to 3.37)	1.00 [Reference]	0.65 (0.36 to 1.20)
**All other causes**				
Events, No.	2,236	380	2,117	499
Crude incidence per 1000 person-years	0.83	1.30	0.83	1.10
HR (95% CI) [a]	1.00 [Reference]	1.38 (1.23 to 1.55)	1.00 [Reference]	0.93 (0.84 to 1.02)

[a] Models were adjusted for age (continuous), history of infertility (yes, no [reference]), BMI at age 18 years (<18.5, 18.5-24.9 [reference], 25-29.9, 20-34.9, ≥35 kg/m^2), menstrual cycle length at age 18-22 years (<26, 26-31 [reference], 32-50, or ≥50 days or too irregular to estimate), age at menarche (<12 [reference], 12, 13, or ≥14 years of age), as well as time-varying non-aspirin NSAID use (yes, no [reference]), aspirin use (yes, no [reference]), oral contraceptive use (current/former, no [reference]), postmenopausal hormone therapy (never [reference], past, current), BMI (<24.9 [reference], 25-29.9, 30-34.9, or ≥35 kg/m^2), smoking status (never [reference], former, current 1-34 cigarettes/day, or current ≥35 cigarettes/day), physical activity (0 [reference]), 0.1-1.0, 1.1-2.4, 2.5-5.9, or ≥6 h/week), and Alternative Healthy Eating Index 2010 diet quality scores (quintiles, with the lowest quintile [reference] representing the least healthy diet).

Table S13. Sensitivity analyses for the association of endometriosis and uterine fibroids with risk of premature mortality by excluding women who died within 5 years since the diagnosis of endometriosis or uterine fibroids (NHSII, 1989-2019).

Cause-specific mortality	Laparoscopically confirmed endometriosis		Ultrasound- or hysterectomy-confirmed uterine fibroids	
	No	Yes	No	Yes
**All death**				
Events, No.	3,717	566	3,483	800
Crude incidence per 1000 person-years	1.38	1.94	1.37	1.77
HR (95% CI) [a]	1.00 [Reference]	1.29 (1.17 to 1.41)	1.00 [Reference]	0.96 (0.88 to 1.03)
**CVD**				
Events, No.	264	33	238	59
Crude incidence per 1000 person-years	0.10	0.11	0.09	0.13
HR (95% CI) [a]	1.00 [Reference]	1.12 (0.77 to 1.64)	1.00 [Reference]	1.06 (0.79 to 1.43)
**Cancer**				
Events, No.	1,250	167	1,141	276
Crude incidence per 1000 person-years	0.46	0.57	0.45	0.61
HR (95% CI) [a]	1.00 [Reference]	1.19 (1.01 to 1.41)	1.00 [Reference]	1.08 (0.95 to 1.24)
**Respiratory diseases**				
Events, No.	73	17	77	13
Crude incidence per 1000 person-years	0.03	0.06	0.03	0.03
HR (95% CI) [a]	1.00 [Reference]	1.95 (1.11 to 3.41)	1.00 [Reference]	0.67 (0.37 to 1.23)
**All other causes**				
Events, No.	2,130	349	2,027	452
Crude incidence per 1000 person-years	0.79	1.19	0.80	1.00
HR (95% CI) [a]	1.00 [Reference]	1.34 (1.18 to 1.50)	1.00 [Reference]	0.89 (0.80 to 0.99)

[a] Models were adjusted for age (continuous), history of infertility (yes, no [reference]), BMI at age 18 years (<18.5, 18.5-24.9 [reference], 25-29.9, 20-34.9, ≥35 kg/m^2), menstrual cycle length at age 18-22 years (<26, 26-31 [reference], 32-50, or ≥50 days or too irregular to estimate), age at menarche (<12 [reference], 12, 13, or ≥14 years of age), as well as time-varying non-aspirin NSAID use (yes, no [reference]), aspirin use (yes, no [reference]), oral contraceptive use (current/former, no [reference]), postmenopausal hormone therapy (never [reference], past, current), BMI (<24.9 [reference], 25-29.9, 30-34.9, or ≥35 kg/m^2), smoking status (never [reference], former, current 1-34 cigarettes/day, or current ≥35 cigarettes/day), physical activity (0 [reference]), 0.1-1.0, 1.1-2.4, 2.5-5.9, or ≥6 h/week), and Alternative Healthy Eating Index 2010 diet quality scores (quintiles, with the lowest quintile [reference] representing the least healthy diet).

Table S14. Sensitivity analyses for the association of endometriosis and uterine fibroids with risk of premature mortality, with adjustment for baseline medicine intake and behavioural factors (n=110,091; NHSII, 1989-2019).

Cause-specific mortality	Laparoscopically confirmed endometriosis		Ultrasound- or hysterectomy-confirmed uterine fibroids	
	No	Yes	No	Yes
**All death**				
Events, No.	3,770	586	3,498	858
Crude incidence per 1000 person-years	1.40	2.01	1.38	1.90
HR (95% CI) [a]	1.00 [Reference]	1.28 (1.17 to 1.41)	1.00 [Reference]	0.99 (0.91 to 1.07)
**CVD**				
Events, No.	270	34	239	65
Crude incidence per 1000 person-years	0.10	0.12	0.09	0.14
HR (95% CI) [a]	1.00 [Reference]	1.09 (0.75 to 1.59)	1.00 [Reference]	1.12 (0.84 to 1.49)
**Cancer**				
Events, No.	1,282	177	1,148	311
Crude incidence per 1000 person-years	0.47	0.61	0.45	0.69
HR (95% CI) [a]	1.00 [Reference]	1.21 (1.02 to 1.42)	1.00 [Reference]	1.18 (1.03 to 1.35)
**Respiratory diseases**				
Events, No.	73	17	77	13
Crude incidence per 1000 person-years	0.03	0.06	0.03	0.03
HR (95% CI) [a]	1.00 [Reference]	1.92 (1.09 to 3.37)	1.00 [Reference]	0.66 (0.36 to 1.21)
**All other causes**				
Events, No.	2,145	358	2,034	469
Crude incidence per 1000 person-years	0.79	1.23	0.80	1.04
HR (95% CI) [a]	1.00 [Reference]	1.33 (1.18 to 1.49)	1.00 [Reference]	0.89 (0.80 to 0.98)

[a] Models were adjusted for age (continuous), history of infertility (yes, no [reference]), BMI at age 18 years (<18.5, 18.5-24.9 [reference], 25-29.9, 20-34.9, ≥35 kg/m^2), menstrual cycle length at age 18-22 years (<26, 26-31 [reference], 32-50, or ≥50 days or too irregular to estimate), age at menarche (<12 [reference], 12, 13, or ≥14 years of age), postmenopausal hormone therapy (never [reference], past, current), baseline BMI (<24.9 [reference], 25-29.9, 30-34.9, or ≥35 kg/m^2), baseline smoking status (never [reference], former, current 1-34 cigarettes/day, or current ≥35 cigarettes/day), baseline physical activity (0 [reference]), 0.1-1.0, 1.1-2.4, 2.5-5.9, or ≥6 h/week), baseline Alternative Healthy Eating Index 2010 diet quality scores (quintiles, with the lowest quintile [reference] representing the least healthy diet), baseline non-aspirin NSAID use (yes, no [reference]), baseline aspirin use (yes, no [reference]), and baseline oral contraceptive use (current/former, no [reference]).

Table S15. Sensitivity analyses for the association of endometriosis and uterine fibroids with risk of premature mortality, with additional adjustment for race/ethnicity (n=110,091; NHSII, 1989-2019).

Cause-specific mortality	Laparoscopically confirmed endometriosis		Ultrasound- or hysterectomy-confirmed uterine fibroids	
	No	Yes	No	Yes
**All death**				
Events, No.	3,770	586	3,498	858
Crude incidence per 1000 person-years	1.40	2.01	1.38	1.90
HR (95% CI) [a]	1.00 [Reference]	1.31 (1.20 to 1.44)	1.00 [Reference]	1.02 (0.95 to 1.11)
**CVD**				
Events, No.	270	34	239	65
Crude incidence per 1000 person-years	0.10	0.12	0.09	0.14
HR (95% CI) [a]	1.00 [Reference]	1.13 (0.77 to 1.63)	1.00 [Reference]	1.16 (0.87 to 1.55)
**Cancer**				
Events, No.	1,282	177	1,148	311
Crude incidence per 1000 person-years	0.47	0.61	0.45	0.69
HR (95% CI) [a]	1.00 [Reference]	1.22 (1.04 to 1.44)	1.00 [Reference]	1.22 (1.07 to 1.38)
**Respiratory diseases**				
Events, No.	73	17	77	13
Crude incidence per 1000 person-years	0.03	0.06	0.03	0.03
HR (95% CI) [a]	1.00 [Reference]	1.95 (1.11 to 3.42)	1.00 [Reference]	0.67 (0.36 to 1.22)
**All other causes**				
Events, No.	2,145	358	2,034	469
Crude incidence per 1000 person-years	0.79	1.23	0.80	1.04
HR (95% CI) [a]	1.00 [Reference]	1.36 (1.21 to 1.53)	1.00 [Reference]	0.92 (0.83 to 1.02)

[a] Models were adjusted for age (continuous), race/ethnicity (non-Hispanic White, other [reference]), history of infertility (yes, no [reference]), BMI at age 18 years (<18.5, 18.5-24.9 [reference], 25-29.9, 20-34.9, ≥35 kg/m^2), menstrual cycle length at age 18-22 years (<26, 26-31 [reference], 32-50, or ≥50 days or too irregular to estimate), age at menarche (<12 [reference], 12, 13, or ≥14 years of age), as well as time-varying non-aspirin NSAID use (yes, no [reference]), aspirin use (yes, no [reference]), oral contraceptive use (current/former, no [reference]), postmenopausal hormone therapy (never [reference], past, current), BMI (<24.9 [reference], 25-29.9, 30-34.9, or ≥35 kg/m^2), smoking status (never [reference], former, current 1-34 cigarettes/day, or current ≥35 cigarettes/day), physical activity (0 [reference]), 0.1-1.0, 1.1-2.4, 2.5-5.9, or ≥6 h/week), and Alternative Healthy Eating Index 2010 diet quality scores (quintiles, with the lowest quintile [reference] representing the least healthy diet).

Table S16. Sensitivity analyses for the association of endometriosis with the risk of premature mortality by including endometriosis cases both with and without laparoscopic confirmation (n=110,111; NHSII, 1989-2019).

Cause-specific mortality	Endometriosis	
	No	Yes
**All death**		
Events, No.	3,644	713
Crude incidence per 1000 person-years	1.38	2.01
HR (95% CI) [a]	1.00 [Reference]	1.28 (1.17 to 1.39)
**CVD**		
Events, No.	265	39
Crude incidence per 1000 person-years	0.10	0.11
HR (95% CI) [a]	1.00 [Reference]	0.99 (0.70 to 1.41)
**Cancer**		
Events, No.	1,240	219
Crude incidence per 1000 person-years	0.47	0.62
HR (95% CI) [a]	1.00 [Reference]	1.22 (1.05 to 1.42)
**Respiratory diseases**		
Events, No.	72	18
Crude incidence per 1000 person-years	0.03	0.05
HR (95% CI) [a]	1.00 [Reference]	1.55 (0.89 to 2.68)
**All other causes**		
Events, No.	2,067	437
Crude incidence per 1000 person-years	0.78	1.23
HR (95% CI) [a]	1.00 [Reference]	1.33 (1.19 to 1.48)

[a] Models were adjusted for age (continuous), history of infertility (yes, no [reference]), BMI at age 18 years (<18.5, 18.5-24.9 [reference], 25-29.9, 20-34.9, ≥35 kg/m^2), menstrual cycle length at age 18-22 years (<26, 26-31 [reference], 32-50, or ≥50 days or too irregular to estimate), age at menarche (<12 [reference], 12, 13, or ≥14 years of age), as well as time-varying non-aspirin NSAID use (yes, no [reference]), aspirin use (yes, no [reference]), oral contraceptive use (current/former, no [reference]), postmenopausal hormone therapy (never [reference], past, current), BMI (<24.9 [reference], 25-29.9, 30-34.9, or ≥35 kg/m^2), smoking status (never [reference], former, current 1-34 cigarettes/day, or current ≥35 cigarettes/day), physical activity (0 [reference]), 0.1-1.0, 1.1-2.4, 2.5-5.9, or ≥6 h/week), and Alternative Healthy Eating Index 2010 diet quality scores (quintiles, with the lowest quintile [reference] representing the least healthy diet).

Table S17. Sensitivity analyses for the association of uterine fibroids with the risk of premature mortality by including uterine fibroid cases both with and without ultrasound or hysterectomy confirmation (n=110,096; NHSII, 1989-2019).

Cause-specific mortality	Uterine fibroids	
	No	Yes
**All death**		
Events, No.	3,393	965
Crude incidence per 1000 person-years	1.37	1.84
HR (95% CI) [a]	1.00 [Reference]	1.00 (0.93 to 1.08)
**CVD**		
Events, No.	235	69
Crude incidence per 1000 person-years	0.09	0.13
HR (95% CI) [a]	1.00 [Reference]	1.08 (0.81 to 1.43)
**Cancer**		
Events, No.	1,116	344
Crude incidence per 1000 person-years	0.45	0.66
HR (95% CI) [a]	1.00 [Reference]	1.15 (1.02 to 1.31)
**Respiratory diseases**		
Events, No.	75	15
Crude incidence per 1000 person-years	0.03	0.03
HR (95% CI) [a]	1.00 [Reference]	0.68 (0.38 to 1.20)
**All other causes**		
Events, No.	1,967	537
Crude incidence per 1000 person-years	0.79	1.02
HR (95% CI) [a]	1.00 [Reference]	0.93 (0.84 to 1.02)

[a] Models were adjusted for age (continuous), history of infertility (yes, no [reference]), BMI at age 18 years (<18.5, 18.5-24.9 [reference], 25-29.9, 20-34.9, ≥35 kg/m^2), menstrual cycle length at age 18-22 years (<26, 26-31 [reference], 32-50, or ≥50 days or too irregular to estimate), age at menarche (<12 [reference], 12, 13, or ≥14 years of age), as well as time-varying non-aspirin NSAID use (yes, no [reference]), aspirin use (yes, no [reference]), oral contraceptive use (current/former, no [reference]), postmenopausal hormone therapy (never [reference], past, current), BMI (<24.9 [reference], 25-29.9, 30-34.9, or ≥35 kg/m^2), smoking status (never [reference], former, current 1-34 cigarettes/day, or current ≥35 cigarettes/day), physical activity (0 [reference]), 0.1-1.0, 1.1-2.4, 2.5-5.9, or ≥6 h/week), and Alternative Healthy Eating Index 2010 diet quality scores (quintiles, with the lowest quintile [reference] representing the least healthy diet).

Table S18. Robustness to unmeasured confounding (E-values[a]) for assessing the associations between endometriosis and uterine fibroids with risk of all-cause and cause-specific premature mortality (n=110,091; NHSII, 1989-2019).

Cause-specific mortality	Laparoscopically confirmed endometriosis		Ultrasound- or hysterectomy- confirmed uterine fibroids	
	E-value Point Estimate	Lower CI for E-value	E-value Point Estimate	Lower CI for E-value
**All death**	1.95	1.69	1.16	1.00
**CVD**	1.51	1.00	1.59	1.00
**Cancer**	1.74	1.24	1.74	1.34
**Respiratory diseases**	3.31	1.46	2.35	1.00
**All other causes**	2.06	1.71	1.39	1.00

Abbreviations: CI, confidence interval.

[a] We calculated E-values using the publicly available online E-value calculator (https://www.hsph.harvard.edu/tyler-vanderweele/tools-and-tutorials/).

E-values for 1) the point estimate and 2) the limit of the 95% confidence interval (CI) closest to the null (i.e. the lower limit for the above CIs) represent the magnitude of the association that an unmeasured confounder would have to have with both the exposure and outcome, above and beyond measured confounding, to 1) explain away the observed association and 2) render the observed association no longer statistically significant, respectively.
